# Systematics of South American snail-eating snakes (Serpentes, Dipsadini), with the description of five new species from Ecuador and Peru

**DOI:** 10.3897/zookeys.766.24523

**Published:** 2018-06-14

**Authors:** Alejandro Arteaga, David Salazar-Valenzuela, Konrad Mebert, Nicolás Peñafiel, Gabriela Aguiar, Juan C. Sánchez-Nivicela, R. Alexander Pyron, Timothy J. Colston, Diego F. Cisneros-Heredia, Mario H. Yánez-Muñoz, Pablo J. Venegas, Juan M. Guayasamin, Omar Torres-Carvajal

**Affiliations:** 1 Richard Gilder Graduate School, American Museum of Natural History, New York, USA; 2 Department of Herpetology, American Museum of Natural History, New York, USA; 3 Tropical Herping, Quito, Ecuador; 4 Centro de Investigación de la Biodiversidad y Cambio Climático (BioCamb), Facultad de Ciencias de Medio Ambiente, Ingeniería en Biodiversidad y Recursos Genéticos, Universidad Tecnológica Indoamérica, Quito, Ecuador; 5 Universidade Estadual de Santa Cruz, Departamento de Ciências Biológicas, Ilhéus, Brazil; 6 Laboratorio de Herpetología, Museo de Zoología de la Universidad del Azuay, Cuenca, Ecuador; 7 Department of Biological Sciences, The George Washington University, Washington, D.C., USA; 8 Department of Vertebrate Zoology, National Museum of Natural History, Smithsonian Institution, Washington, D.C., USA; 9 Universidad San Francisco de Quito USFQ, Colegio de Ciencias Biológicas y Ambientales, Instituto de Zoología Terrestre & Museo de Zoología, Quito, Ecuador; 10 División de Herpetología, Instituto Nacional de Biodiversidad (INABIO), Quito, Ecuador; 11 King’s College London, Department of Geography, London, UK; 12 División de Herpetología-Centro de Ornitología y Biodiversidad (CORBIDI), Lima, Peru; 13 Universidad San Francisco de Quito (USFQ), Colegio de Ciencias Biológicas y Ambientales, Laboratorio de Biología Evolutiva, campus Cumbayá, Quito, Ecuador; 14 Museo de Zoología, Escuela de Ciencias Biológicas, Pontificia Universidad Católica del Ecuador, Quito, Ecuador

**Keywords:** Dipsadini, *Dipsas*, Ecuador, new species, Peru, phylogeny, *Sibon*, *Sibynomorphus*, snail-eating snakes, systematics

## Abstract

A molecular phylogeny of the Neotropical snail-eating snakes (tribe Dipsadini) is presented including 43 (24 for the first time) of the 77 species, sampled for both nuclear and mitochondrial genes. Morphological and phylogenetic support was found for four new species of *Dipsas* and one of *Sibon*, which are described here based on their unique combination of molecular, meristic, and color pattern characteristics. *Sibynomorphus* is designated as a junior subjective synonym of *Dipsas*. *Dipsas latifrontalis* and *D. palmeri* are resurrected from the synonymy of *D. peruana*. *Dipsas latifasciata* is transferred from the synonymy of *D. peruana* to the synonymy of *D. palmeri*. A new name, *D. jamespetersi*, is erected for the taxon currently known as *Sibynomorphus petersi*. Re-descriptions of *D. latifrontalis* and *D. peruana* are presented, as well as the first photographic voucher of an adult specimen of *D. latifrontalis*, along with photographs of all known Ecuadorian Dipsadini species. The first country record of *D. variegata* in Ecuador is provided and *D. oligozonata* removed from the list of Peruvian herpetofauna. With these changes, the number of Dipsadini reported in Ecuador increases to 22, 18 species of *Dipsas* and four of *Sibon*.

## Introduction

With 70 currently recognized species (Table 1), the snail-eaters (tribe Dipsadini) are among the most diverse groups of arboreal snakes (Wallach et al. 2014; Uetz et al. 2016). Some authors have suggested that their tree-dwelling lifestyle and specialized diet resulted this large an adaptive radiation (e.g., MacCulloch and Lathrop 2004; Sheehy 2012). In the last decade, the limits of the tribe have been redefined to include five genera (*Dipsas*, *Plesiodipsas*, *Sibon*, *Sibynomorphus*, and *Tropidodipsas*; Harvey et al. 2008), but recent studies suggest that not all of them are monophyletic (Sheehy 2012; Figueroa et al. 2016). Consequently, the limits between genera, species, and species groups appear to be poorly defined, and in need of revision for a robust and stable taxonomy.

One of the first modern attempts to clarify the taxonomy and summarize knowledge on the tribe Dipsadini was published by Peters (1960). Peters considered Dipsadini to include the genera *Dipsas*, *Sibon* and *Sibynomorphus*. Later, Zaher (1999) and Harvey et al. (2008) added *Tropidodipsas* and *Plesiodipsas* in the tribe. Peters also created seven species groups within *Dipsas*, three within *Sibon* (Table 1), and recognized *D. boettgeri*, *D. latifrontalis*, *D. latifasciata*, *D. polylepis*, and *D. peruana* as distinct species based on coloration and lepidosis. However, he considered *D. palmeri* and *D. praeornata* to be synonyms of *D. latifrontalis*.

After Peters, several authors continued to address the systematics of the group (Downs 1961, Hoge 1964, Peters and Orejas-Miranda 1970, Kofron 1982, Orcés and Almendáriz 1987, Porto and Fernandes 1996, Fernandes et al. 1998, Fernandes et al. 2002, Cadle and Myers 2003, Passos et al. 2004, Passos et al. 2005, Cadle 2005, Cadle 2007, Harvey 2008, Harvey and Embert 2008, Harvey et al. 2008). Of these, the works by Cadle and Myers (2003), Cadle (2007), Harvey (2008), and Harvey and Embert (2008) are worth addressing further because they focused on Ecuadorian species for which there is still taxonomic uncertainty. Cadle and Myers (2003) removed *D. variegata* from the herpetofauna of Ecuador, since previous records were based on museum misidentifications. Cadle (2007) reviewed the status of species of *Sibynomorphus* in Ecuador and Peru, and referred three additional specimens (AMNH 110587, BMNH 1935.11.3.108, and MUSM 2192) to *S. oligozonatus*, including the first country record for Peru. Cadle (2005) also reviewed three specimens of *D. gracilis* collected in Peru; however, Harvey (2008) concluded that only one of them corresponded to *D. gracilis*. In the same work, Harvey also redefined Peters’ (1960) species groups (Table 1). Finally, Harvey and Embert (2008) transferred *D. boettgeri*, *D. latifrontalis*, and *D. polylepis* to the synonymy of *D. peruana*, based on both the difficulty of segregating these species using morphological characters and their “more or less continuous distribution along the eastern slopes of the Andes”.

Here, we combine morphological analysis and molecular phylogenetics to revise generic and species limits within Dipsadini. We combine all available molecular sampling with new samples from Ecuador, Peru, Brazil and Costa Rica, and find support for five new species, as well as a number of changes to the geographic distribution of several Andean species.

**Table 1.** Taxonomy of Dipsadini prior to this paper.

Genus	Group	Species	Authority	Reference
*Dipsas*	*D. articulata*	*D. articulata*	Cope, 1868	Harvey 2008
		*D. bicolor*	Günther, 1895	Peters 1960
		*D. brevifacies*	Cope, 1866	Harvey 2008
		*D. gaigeae*	Oliver, 1837	Harvey 2008
		*D. gracilis*	Boulenger, 1902	Harvey 2008
		*D. maxillaris*	Werner, 1910	Peters 1960
		*D. tenuissima*	Taylor, 1954	Harvey 2008
		*D. viguieri*	Bocourt, 1884	Harvey 2008
	*D. catesbyi*	*D. catesbyi*	Sentzen, 1796	Harvey 2008
		*D. copei*	Günther, 1872	Harvey 2008
		*D. pavonina*	Schlegel, 1837	Harvey 2008
	*D. elegans*	*D. elegans*	Boulenger, 1896	Harvey 2008
		*D. ellipsifera*	Boulenger, 1898	Harvey 2008
		*D. oreas*	Cope, 1868	Harvey 2008
	*D. incerta*	*D. alternans*	Fischer, 1885	Harvey 2008
		*D. incerta*	Jan, 1863	Harvey 2008
		*D. praeornata*	Werner, 1909	Harvey 2008
		*D. sazimai*	Fernandes et al., 2010	Fernandes et al. 2010
	*D. indica*	*D. bucephala*	Shaw, 1802	Harvey 2008
		*D. cisticeps*	Boettger, 1885	Harvey 2008
		*D. indica*	Laurenti, 1768	Harvey 2008
	*D. pratti*	*D. baliomelas*	Harvey, 2008	Harvey 2008
		*D. chaparensis*	Reynolds & Foster, 1992	Harvey 2008
		*D. peruana*	Boettger, 1898	Harvey 2008
		*D. pratti*	Boulenger, 1897	Harvey 2008
		*D. sanctijoannis*	Boulenger, 1911	Harvey 2008
		*D. schunkii*	Boulenger, 1908	Harvey 2008
	*D. temporalis*	*D. pakaraima*	MacCulloch & Lathrop, 2004	Harvey 2008
		*D. temporalis*	Werner, 1909	Harvey 2008
		*D. vermiculata*	Peters, 1960	Harvey 2008
	*D. variegata*	*D. albifrons*	Sauvage, 1884	Harvey 2008
		*D. andiana*	Boulenger, 1896	Harvey 2008
		*D. nicholsi*	Dunn, 1933	Harvey 2008
		*D. trinitatis*	Parker, 1926	Harvey 2008
		*D. variegata*	Duméril et al., 1854	Harvey 2008
*Plesiodipsas*	Unassigned	*P. perijanensis*	Aleman, 1953	–
*Sibon*	*S. annulatus*	*S. annulatus*	Günther, 1872	Savage 2002
		*S. anthracops*	Cope, 1868	Savage 2002
		*S. dimidiatus*	Günther, 1872	Savage 2002
		*S. lamari*	Solórzano, 2001	Solórzano 2001
		*S. linearis*	Pérez-Higareda et al., 2002	Pérez-Higareda et al. 2002
		*S. manzanaresi*	McCranie, 2007	McCranie 2007
		*S. merendonensis*	Rovito et al., 2012	Rovito et al. 2012
		*S. miskitus*	McCranie, 2006	McCranie 2006
		*S. sanniolus*	Cope, 1866	Savage 2002
*Sibon*	*S. argus*	*S. argus*	Cope, 1875	Savage 2002
		*S. longifrenis*	Stejneger, 1909	Savage 2002
	*S. nebulatus*	*S. carri*	Shreve, 1951	Peters 1960
		*S. dunni*	Peters, 1957	Savage 2002
		*S. nebulatus*	Linnaeus, 1758	Savage 2002
	Unassigned	*S. noalamina*	Lotzkat et al., 2012	–
		*S. perissostichon*	Köhler et al., 2010	–
*Sibynomorphus*	Unassigned	*S. lavillai*	Scrocchi et al., 1993	–
		*S. mikanii*	Schlegel, 1837	–
		*S. neuwiedi*	Ihering, 1911	–
		*S. oligozonatus*	Orcés & Almendáriz, 1989	–
		*S. oneilli*	Rossman & Thomas, 1979	–
		*S. petersi*	Orcés & Almendáriz, 1989	–
		*S. turgidus*	Cope, 1868	–
		*S. vagrans*	Dunn, 1923	–
		*S. vagus*	Jan, 1863	–
		*S. ventrimaculatus*	Boulenger, 1885	–
		*S. williamsi*	Carillo de Espinoza, 1974	–
*Tropidodipsas*	*T. fasciata*	*T. fasciata*	Günther, 1858	Kofron 1987
		*T. philippii*	Jan, 1863	Kofron 1987
	*T. sartorii*	*T. annulifera*	Boulenger, 1894	Kofron 1988
		*T. sartorii*	Cope, 1863	Kofron 1988
		*T. zweifeli*	Liner & Wilson, 1970	Kofron 1988
	Unassigned	*T. fischeri*	Boulenger, 1894	–
		*T. repleta*	Smith et al., 2005	–

## Materials and methods

### Ethics statement

This study was carried out in strict accordance with the guidelines for use of live amphibians and reptiles in field research (Beaupre et al. 2004) compiled by the American Society of Ichthyologists and Herpetologists (ASIH), the Herpetologists’ League (HL) and the Society for the Study of Amphibians and Reptiles (SSAR). All procedures with animals (see below) were reviewed by the Ministerio de Ambiente del Ecuador (MAE) and specifically approved as part of obtaining the following field permits for research and collection: MAE-DNB-CM-2015-0017 (granted to Universidad Tecnológica Indoamérica), 018-IC-FAU-DNBAP/MA, 010-IC-FAU-DNBAPVS/MA, 004-IC-FAU/FLO-DPZCH-MA (granted to Museo Ecuatoriano de Ciencias Naturales del Instituto Nacional de Biodiversidad) and 001-10 IC-FAU-DNB/MA, 001-11 IC-FAU-DNB/MA, 002-16 IC-FAU-DNB/MA, 003-15 IC-FAU-DNB/MA, 003-17 IC-FAU-DNB/MA, 005-14 IC-FAU-DNB/MA, 008-09 IC-FAU-DNB/MA, MAE-DNB-ARRGG-CM-2014-0002 (granted to Pontificia Universidad Católica del Ecuador). Specimens were euthanized with 20% benzocaine, fixed in 10% formalin or 70% ethanol, and stored in 70% ethanol. Museum vouchers were deposited at Museo de Zoología of the Universidad Tecnológica Indoamérica (MZUTI), Museo de Zoología (QCAZ) of Pontificia Universidad Católica del Ecuador, Museo de Zoología (ZSFQ) of Universidad San Francisco de Quito, División de Herpetología (DHMECN) of Instituto Nacional de Biodiversidad and Coleção Herpetológica da UnB (CHUNB).

### Common names

Criteria for common name designation are as proposed by Caramaschi et al. (2006), as modified by Coloma and Guayasamin (2011–2017), and are as follows (in order of importance): (i) the etymological intention (implicit or explicit) that the authors used when naming the species (specific epithet); (ii) a common name that is already widely used in the scientific literature; (iii) a common name that has an important ancestral or cultural meaning; (iv) a common name based on any distinctive aspect of the species (distribution, morphology, behavior, etc.).

### Sampling

**Figure 1.**
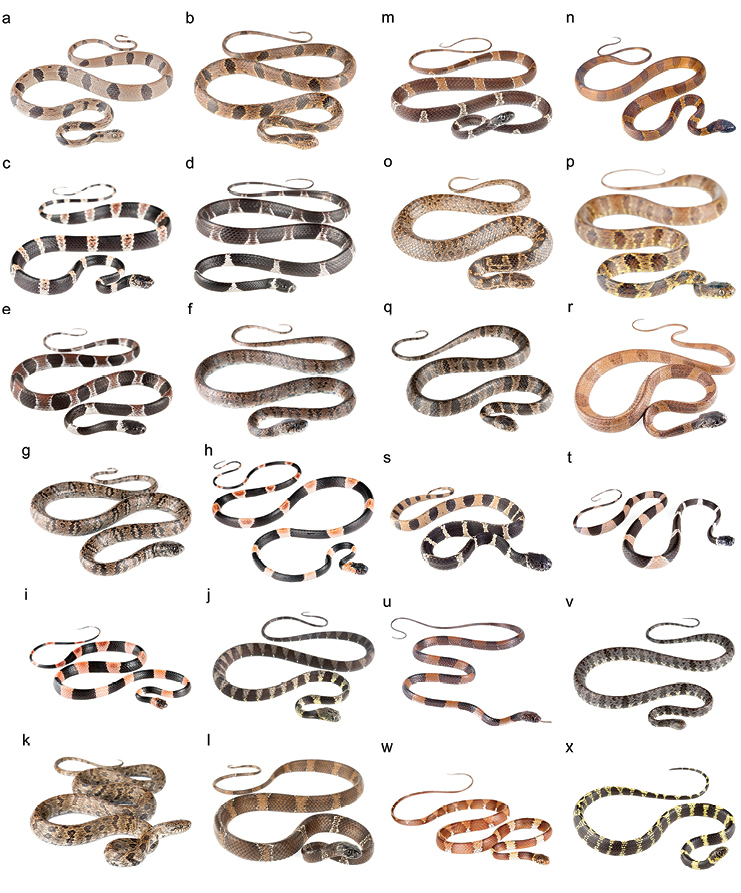
Photographs of some species of *Dipsas* in life: **a** *D. andiana* MZUTI 5413 from Bilsa, province of Esmeraldas, Ecuador **b** *D. andiana* from Mindo, province of Pichincha, Ecuador **c** *D. bobridgelyi* MZUTI 5414 from Buenaventura, Province of El Oro, Ecuador **d** *D. catesbyi* from Gareno, province of Napo, Ecuador **e** *D. catesbyi* from Gareno, province of Napo, Ecuador **f** *D. elegans* from Calacalí–Mindo, province of Pichincha, Ecuador **g** *D. ellipsifera* from Pimampiro, province of Imbabura, Ecuador **h** *D. gracilis* from Canandé, province of Esmeraldas, Ecuador **i** *D. gracilis* from Mashpi, province of Pichincha, Ecuador **j** *D. indica* from Gareno, province of Napo, Ecuador **k** *D. jamespetersi* AMARU 1123 from province of Azuay, Ecuador **l** *D. klebbai* from El Chaco, province of Napo, Ecuador **m** *D. klebbai* from El Chaco, province of Napo, Ecuador **n** *D. latifrontalis* from San Isidro, state of Mérida, Venezuela **o** *D. oligozonata* from Poetate, province of Azuay, Ecuador **p** *D. oreas* MZUTI 5414 from Buenaventura, province of El Oro, Ecuador **q** *D. oreas* from Poetate–Corraleja, province of Azuay, Ecuador **r** *D. palmeri* from Agoyán, province of Tungurahua, Ecuador **s** *D. palmeri* MZUTI 4975 from Reserva San Francisco, province of Zamora, Ecuador **t** *D. pavonina* from Maycu, province of Zamora, Ecuador **u** *D. temporalis* from Colombia **v** *D. variegata* from Gareno, province of Napo, Ecuador **w** *D. vermiculata* from Miazi, province of Zamora, Ecuador, and **x** *D. vermiculata* from Narupa, province of Napo, Ecuador.

**Figure 2.**
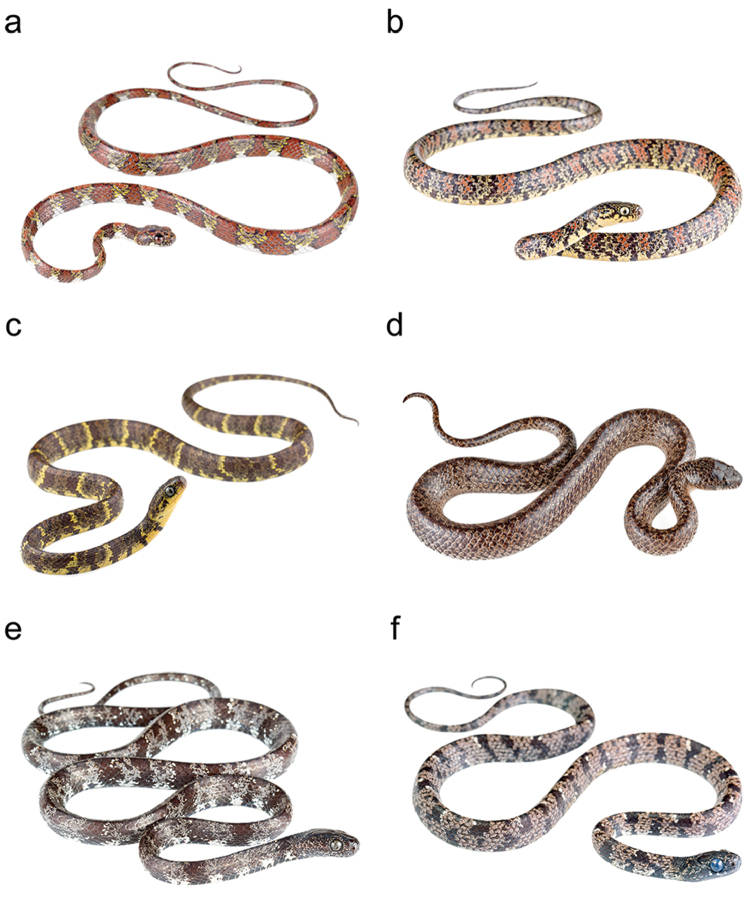
Photographs of some species of *Sibon* in life: **a** *S. annulatus* from Verdecanandé, province of Esmeraldas, Ecuador **b** *Sibon bevridgelyi* MZUA.RE.0424 from Palmales Nuevo, province of El Oro, Ecuador **c** *S. bevridgelyi* MZUTI 3269 from Buenaventura, province of El Oro, Ecuador **d** *S. dunni* CAMPO 533 from Pimampiro, province of Imbabura, Ecuador **e** *S. nebulatus* from Milpe, province of Pichincha, Ecuador, and **f** *S. nebulatus* from Canandé, province of Esmeraldas, Ecuador.

Tissue samples from 85 individuals representing 28 species (including five new species described here) were sampled from Ecuador, Peru, Guatemala, Costa Rica, Nicaragua, Brazil, and Mexico. All specimens included in the genetic analyses were morphologically identified according to Arteaga et al. (2013), Cadle (2005), Cadle (2007), Cadle and Myers (2003), Duellman (1978), Harvey (2008), Harvey and Embert (2008), Peters (1957) and Savage (2002). We created photo vouchers (Figs 1, 2) for all Ecuadorian species of Dipsadini. We generated sequence data for samples marked with an asterisk under Appendix 1, which includes museum vouchers at MZUTI, QCAZ, Museo de Zoología de la Universidad del Azuay (MZUA), División de Herpetología del Instituto Nacional de Biodiversidad (DHMECN), Museum of Vertebrate Zoology at Berkeley (MVZ), Bioparque Amaru Cuenca (AMARU), Coleção Herpetológica da UnB (CHUNB), Museo de Zoología de la Universidad San Francisco de Quito (ZSFQ), and Centro de Ornitología y Biodiversidad (CORBIDI), along with individuals not accessioned in musem collections (CAMPO, JMG and TJC).

### Laboratory techniques

Genomic DNA was extracted from 96% ethanol-preserved tissue samples (liver, muscle tissue or scales) using either a guanidinium isothiocyanate extraction protocol, or a modified salt precipitation method based on the Puregene DNA purification kit (Gentra Systems). We amplified the 16S gene using primer pairs 16Sar-L / 16Sbr-H-R from Palumbi et al. (1991) and 16sF.0 (Pellegrino et al. 2001) / 16sR.0 (Whiting et al. 2003). Additionally, the Cytb gene was obtained with primer pairs GLUDG-L (Palumbi et al. 1991) / ATRCB3 (Harvey et al. 2000) and LGL765 (Bickham et al. 1995) / CytbV (Torres-Carvajal et al. 2015), whereas the gene coding for the subunit 4 of the NADH dehydrogenase was amplified with the primers ND4 and Leu developed by Arévalo et al. (1994). The c-mos gene was retrieved with primers S77 and S78 developed by Lawson et al. (2005). PCR reactions contained 2 mM (Cytb and ND4) or 3 mM (16S and c-mos) MgCl_2_, 200 µM dNTP mix, 0.2 µM (16S, Cytb and c-mos) or 0.8 µM (ND4) of each primer and 1.25 U (16S) or 0.625 U (ND4, Cytb and c-mos) Taq DNA Polymerase Recombinant (Thermo Fisher Scientific) in a 25 µL total volume. The nucleotide sequences of the primers and the PCR conditions applied to each primer pair are detailed in Appendix 2. PCR products were cleaned with either ExoSAP-IT (Affymetrix, Cleveland, OH), or Exonuclease I and Alkaline Phosphatase (Illustra ExoProStar by GE Healthcare) before they were sent to Macrogen Inc (Korea) for sequencing. All PCR products were sequenced in both forward and reverse directions with the same primers that were used for amplification. The edited sequences were deposited in GenBank (Appendix 1).

### DNA sequence analyses

A total of 298 DNA sequences were used to build a phylogenetic tree of the tribe Dipsadini, of which 222 were generated during this work and 76 were downloaded from GenBank. Among the new sequences, 103 are 201–520 bp long fragments of the 16S gene, 91 are 586–1,090 bp long fragments of the Cytb gene, 45 are 443–583 bp long fragments of the c-mos gene, 31 are 242–473 bp long fragments of the 12S gene, and 28 are 593–699 bp long fragments of the ND4 gene. New sequences were edited and assembled using the program Geneious ProTM 5.4.7 (Drummond et al. 2010) and aligned with those downloaded from GenBank (Appendix 1) using MAFFT v.7 (Katoh and Standley 2013) under the default parameters in Geneious ProTM 5.4.7. Genes were combined into a single matrix with 11 partitions, one per non-coding gene and three per protein-coding gene corresponding to each codon position. The best partition strategies along with the best-fit models of evolution were obtained in PartitionFinder 2 (Lanfear et al. 2016) under the Bayesian information criterion.

Phylogenetic relationships were assessed under both a Bayesian inference (BI) and a maximum likelihood (ML) approach in MrBayes 3.2.0 (Ronquist and Huelsenbeck 2013) and RAxML v8.2.9 (Stamatakis 2006), respectively. For the ML analysis, nodal support was assessed using the rapid-bootstrapping algorithm with 1000 non-parametric bootstraps. All ML estimates and tests were run under the GTRCAT model, as models available for use in RAxML are limited to variations of the general time-reversible (GTR) model of nucleotide substitution. For the BI analysis, four independent analyses were performed to reduce the chance of converging on a local optimum. Each analysis consisted of 6,666,667 generations and four Markov chains with default heating settings. Trees were sampled every 1,000 generations and 25% of them were arbitrarily discarded as ‘‘burn-in.” The resulting 5,000 saved trees per run were used to calculate posterior probabilities (PP) for each bipartition in a 50% majority-rule consensus tree. We used Tracer 1.6 (Rambaut et al. 2018) to assess convergence and effective sample sizes (ESS) for all parameters. Additionally, we verified that the average standard deviation of split frequencies between chains and the potential scale reduction factor (PSRF) of all the estimated parameters approached values of ≤0.01 and 1, respectively. Genetic distances between new species and their closest morphological relative were calculated using the uncorrected distance matrix in PAUP 4.0 (Swofford 2002). GenBank accession numbers are listed in Appendix 1.

### Morphological data

Terminology for Dipsadini cephalic shields follows proposals by Peters (1960) and Harvey and Embert (2008). Diagnoses and descriptions generally follow Fernandes et al. (2010), and ventral and subcaudal counts follow Dowling (1951). When providing the standard deviation, we use the ± symbol. We examined comparative alcohol-preserved specimens from the herpetology collections at Museo de Zoología de la Universidad Tecnológica Indoamérica (MZUTI), Museum d’Histoire Naturelle de la Ville de Genève (MHNG), Museo de Zoología de la Pontificia Universidad Católica del Ecuador (QCAZ), National Museum of Natural History (USNM), División de Herpetología del Instituto Nacional de Biodiversidad (DHMECN), Museo de Zoología de la Universidad del Azuay (MZUA), American Museum of Natural History (AMNH), Museo de Zoología de la Universidad San Francisco de Quito (ZSFQ), Museum of Natural Science of the Louisiana State University (LSUMZ), Museum of Comparative Zoology of Harvard University (MCZ), Natural History Museum and Biodiversity Research Center of University of Kansas (KU), British Museum of Natural History (BMNH), Museo de Historia Natural de la Escuela Politécnica Nacional (EPN), and Museo de la Universidad Nacional de San Marcos (MUSM) (Table 2). Morphological measurements were taken with measuring tapes to the nearest 1 mm, or with digital calipers to the nearest 0.1 mm. Abbreviations are as follows: snout-vent length (SVL); tail length (TL). Sex was determined by establishing the presence/absence of hemipenes through a subcaudal incision at the base of the tail unless hemipenes were everted.

## Results

### Molecular phylogeny and taxonomic consequences

**Figure 3.**
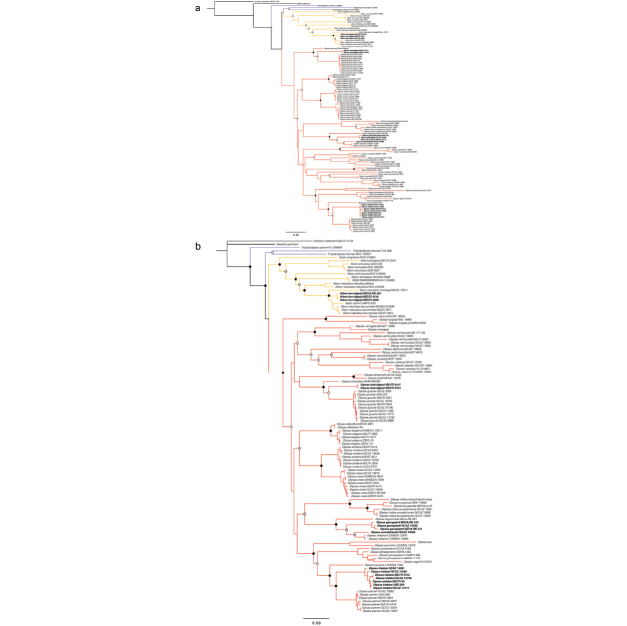
Phylogenetic relationships within Dipsadini derived from analysis of 3,375 bp of DNA (gene fragments 12S, 16S, Cytb, ND4 and c-mos). Support values on intraspecic branches are not shown for clarity. Voucher numbers for sequences are indicated for each terminal when available. **a** Maximum likelihood analysis. Black dots indicate clades with bootstrap values from 90–100%. Grey dots indicate values from 70–89%. White dots indicate values from 50–69% (values <50% not shown) **b** Bayesian inference analysis. Black dots indicate clades with posterior probability values from 95–100%. Grey dots indicate values from 70–94%. White dots indicate values from 50–69% (values <50% not shown).

We consider strong support to be bootstrap values of >70% and posterior probability values >95% following Felsenstein (2004). Overall, there is low support for the relationship between the genera *Dipsas*, *Sibon*, and *Tropidodipsas* (Fig. 3). The genus *Sibynomorphus* is not monophyletic and the included species are nested in four mutually exclusive clades within *Dipsas*. Two of the three included species of *Tropidodipsas*, *T. fischeri*, and *T. fasciata*, form a poorly supported clade, whereas *T. sartorii* is strongly supported as sister lineage to all other included samples of Dipsadini. The genus *Sibon* is monophyletic, and sister to *T. fischeri* and *T. fasciata* in the ML analysis, although this relationship is not strongly supported. In the BI analysis, *Sibon* is sister to *Dipsas*. We excluded *Sibon noalamina* (voucher SMF 91539) from the analyses as the short sequence available in GenBank (gene fragment 16S) represented a rogue taxon that assumed varying phylogenetic positions in the tree collection used to build the consensus tree.

*Sibon longifrenis* is recovered as the sister taxon to all other included species of *Sibon*. Deep intraspecific divergence is found between samples of *S. annulatus* from Central America (MVZ 269290, ADM 0007, ADM 242) and that from Ecuador (MZUTI 3034). The widespread species *S. nebulatus* is paraphyletic with respect to both *S. dunni* and a new species from Ecuador. Nonetheless, within *S. nebulatus*, the included subspecies *S. n. nebulatus* (Linnaeus, 1758) and *S. n. leucomelas* (Boulenger, 1896) are monophyletic, while the single Colombian specimen of *S. n. hartwegi* (Peters 1960) is sister to all other members of the Ecuadorian *S. nebulatus* group. However, posterior probabilites from our genetic data for the formation of monophyletic Ecuadorian clades *S. n. leucomelas*, *S. dunni*, and *Sibon*. sp. are variable, and as low as 48% PP for the node separating *Sibon* sp. from *S. nebulatus leucomelas* and *S. dunni*.

Eight *Sibynomorphus* species were included in the molecular analyses. These are *S. mikanii*, *S. neuwiedi*, *S. oligozonatus*, *S. petersi*, *S. turgidus*, *S. vagus*, *S. ventrimaculatus*, and *S. williamsi*. In the ML analysis, all of them are nested within different *Dipsas* subclades, whereas in the BI analysis, the clade containing *S. mikanii* and *S. turgidus* is not nested within *Dipsas*. Crucially, *Dipsas mikanii* Schlegel, 1837 is the type species of *Sibynomorphus* (Fitzinger, 1843). Thus, we synonymize *Sibynomorphus* with *Dipsas* primarily based on the ML analysis, which mirrors the results of Sheehy (2012).

Based on our transfer of the genus *Sibynomorphus* Fitzinger to the synonymy of *Dipsas*, we propose the following binomial nomenclature for the eleven species traditionally included in the genus *Sibynomorphus*: *Dipsas lavillai* comb. n., *D. mikanii*, *D. neuwiedi* comb. n., *D. oligozonata* comb. nov., *D. oneilli* comb. n., *D. turgida* comb. nov., *D. vagrans* comb. n., *D. vaga* comb. n., *D. ventrimaculata* comb. n., and *D. williamsi* comb. n. However, we refrain from applying *D.* “*petersi*” for *Sibynomorphus petersi* here, because the name *Dipsas* “*indica*” *petersi* (Hoge & Romano, 1975), another taxon and putative species from southeastern Brazil, is often already named as *Dipsas petersi* (e.g., Centeno et al. 2008, Wallach et al. 2014), and this name predates *Sibynomorphus petersi* (Orces & Almendáriz, 1989). Therefore, the latter is now a secondary junior homonym in conflict upon transfer to *Dipsas* Laurenti, and thus requires a replacement name. We therefore erect the name *Dipsas jamespetersi*, which still honors James A. Peters, for the taxon *Sibynomorphus petersi* Orces & Almendariz, 1989.

**Figure 4.**
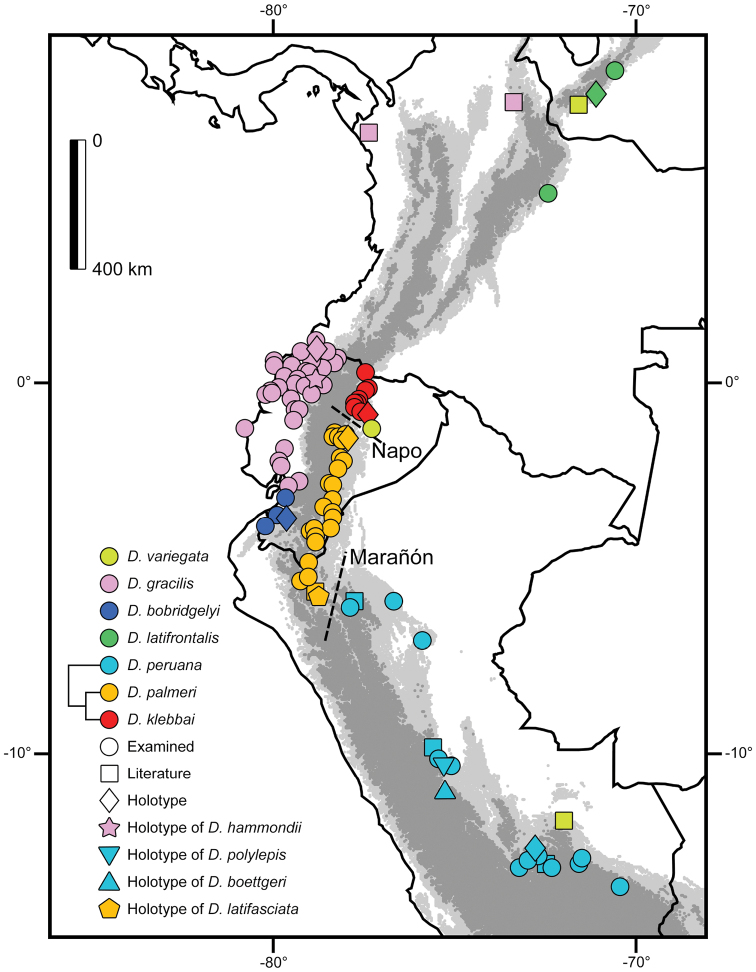
Distribution of various species of *Dipsas*, and potential geographical barriers between taxa.

There are several clades within *Dipsas peruana* sensu lato. One is *D. peruana*, the other is a new species from northern Ecuador, which we describe below, and the third is the lineage corresponding to the population distributed along the Amazonian slopes of the Andes between central Ecuador and northern Peru. Below, we resurrect the name *D. palmeri* (Boulenger, 1912) for this lineage, as the type locality of *D. palmeri* (El Topo, province of Tungurahua, Ecuador) is located within the geographic range of the included samples (Fig. 4) and the holotype agrees in coloration and lepidosis with other specimens (Appendix 3) in the same region that were included in the genetic analyses.

*Dipsas oligozonata* is the strongly supported sister lineage of a clade that includes three species: *D. williamsi* and two new species from western Ecuador and northern Peru, which we describe below. *Dipsas indica* is paraphyletic with respect to *D. bucephala*. *Dipsas jamespetersi* is paraphyletic with respect to a sample of *D. vaga* (KU 219121).

Based on the species included in the phylogenetic analysis, the *Dipsas articulata* and *D. indica* groups, sensu Harvey 2008 (Table 1), are recovered as monophyletic. The other groups included in the phylogenetic analysis (i.e., *catesbyi*, *oreas*, *pratti*, *temporalis* and *variegata*) are not monophyletic. The two included members of the *D. catesbyi* group (i.e., *D. catesbyi* and *D. pavonina*) are not sister taxa. The included members of the *Dipsas oreas* group form a paraphyletic unit, because besides including *D. elegans*, *D. ellipsifera*, and *D. oreas*, this group also includes *D. andiana*, a species that was considered a member of the *D. variegata* group (Harvey 2008, and Table 1). Accordingly, we transfer *D. andiana* to the *D. oreas* group. The two included members of the *D. pratti* group (i.e., *D. peruana* and *D. pratti*) are placed in different branches of the phylogeny. The same is true for the included members of the *D. temporalis* group (i.e., *D temporalis* and *D. vermiculata*), whereby *D. vermiculata* clusters with *D. variegata*, and accordingly we move it into that group. We refrain from merging the *Dipsas temporalis* and *D. pratti* groups because we did not examine the specimens of *D. pratti* included in the analysis (MHUA 14278). We also refrain from assigning further species groups until a more complete taxon sampling is made available.

### New records for Ecuador

One individual (Fig. 1v) of *Dipsas variegata* photographed (not collected) at Gareno Lodge, province of Napo (S1.03559, W77.39864; 336 m), represents the first record of this species in Ecuador (Fig. 4). This individual agrees in coloration with the description of the species provided by Cadle and Myers (2003) and Mebert et al. (submitted), including dorso-lateral blotches/saddles resembling vertically stretched rhomboids or bars, often with a light center or spots, border of blotches being zig-zag shaped and following the outline of adjacent dorsal scales, variably numbered and shaped spots in the interspaces, cephalic blotches lacking yellow borders, and a light-colored eye. It shows also the typical truncated head (see Peters 1960 for description of head truncation) of *D. variegata*, in particular the short, but high preorbital region including an upturned chin, a convex supraocular, narrow and vertically elongated anterior labials (here 2^nd^–6^th^ supralabials), and 15 dorsal scale rows. This *D. variegata* expands the known distribution 1,186 km SW from the nearest localities along the Venezuelan Andes (Natera-Mumaw et al. 2015) and 1,343 km NW from the nearest locality in southeastern Peru (Catenazzi et al. 2013).

### Systematic accounts

We seek here to name or provide re-descriptions only for species that are monophyletic in our molecular phylogeny and share diagnostic features of their coloration pattern and lepidosis. Based on these species delimitation criteria, which follow the general species concept of de Queiroz (2007), we describe four new *Dipsas*, one new *Sibon* and revalidate *D. palmeri* and *D. latifrontalis*.

#### Sibon bevridgelyi sp. n.

Taxon classificationAnimaliaSquamataDipsadidae

http://zoobank.org/E98CD0B9-A101-4693-9529-0AC2134DFECE

Figs 2b , 6 , 7

##### Proposed standard English name.

Bev Ridgely’s Snail-Eater

##### Proposed standard Spanish name.

Caracolera de Bev Ridgely

##### Holotype.

MZUTI 5416 (Figs 6, 7), adult male collected by Matthijs Hollanders on August 01, 2017 at Reserva Buenaventura, province of El Oro, Ecuador (S3.65467, W79.76794; 524 m).

##### Paratypes.

AMNH 22092, adult male collected by George H. Tate on December 01, 1921 at Bucay, province of Guayas, Ecuador (S2.19788, W79.12909; 433 m). CORBIDI 3791, adult male collected by Pablo Venegas and Caroll Landauro on May 07, 2009 at El Caucho, department of Tumbes, Peru (S3.81438, W80.27101; 379 m). CORBIDI 3792, adult female collected by Pablo Venegas and Caroll Landauro on May 07, 2009 at El Caucho, department of Tumbes, Peru (S3.81438, W80.27101, 379 m). CORBIDI 7894, adult female collected by Vilma Durán and Germán Chávez on October 21, 2010 at El Caucho, department of Tumbes, Peru (S3.81844, W80.26856; 478 m). CORBIDI 7994, adult female collected by Pablo Venegas on September 24, 2010 at El Caucho, department of Tumbes, Peru (S3.81244, W80.26716; 481 m). DHMECN 8976, juvenile collected by Michael Harvey and Luis A. Oyagata at Cerro San Sebastián, Parque Nacional Machalilla, province of Manabí, Ecuador (S1.60002, W80.69974, 602 m). DHMECN 9483, adult male collected by Mario Yánez-Muñoz, María Pérez, Miguel Alcoser, Marco Reyes-Puig and Gabriela Bautista in 2012 at the type locality. DHMECN 10061, adult male collected by Manuel Morales, María Perez Lara and Karem López at Reserva Biológica Ayampe, province of Manabí, Ecuador (S1.65417, W80.81333; 43 m). DHMECN 11526, adult of undetermined sex collected by Juan Carlos Sánchez-Nivicela, Karem López, Verónica Urgilés, Bruno Timbe, Elvis Celi and Valentina Posse at Remolino, province of El Oro, Ecuador (S3.56551, W79.91948; 229 m). KU 152205, adult of undetermined sex collected at 30 km E Pasaje, province of Azuay, Ecuador (S3.31439, W79.57970; 561 m). MCZ R-17099, a juvenile of undetermined sex collected at Valle del Chanchán, province of Chimborazo, Ecuador (S2.27383, W79.08735; 697 m). MCZ R-3564, a juvenile of undetermined sex collected by Samuel Walton Garman on January 1, 1875 at Daule River, province of Guayas, Ecuador (S1.87009, W80.00530; 5 m). MZUA.RE.0142, adult female collected by Jose Manuel Falcón at Sarayunga, province of Azuay, Ecuador (S3.31431, W79.58069; 552 m). MZUA.RE.0328, adult male collected by Keyko Cruz on April 04, 2016 at Jauneche, province of Los Ríos, Ecuador (S1.33333, W79.58333; 41 m). MZUA.RE.0424, adult male collected by Fausto Siavichay, Valentina Posse and Xavier Clavijo on June 29, 2017 at 2 km N Palmales Nuevo, province of El Oro, Ecuador (S3.65158, W80.09625; 129 m). MZUTI 3269, adult male collected by Lucas Bustamante on November 07, 2013 at the type locality. QCAZ 14444, adult male collected by Fernando Ayala, Steven Poe and Chris Anderson on January 10, 2016 at Proyecto Minas San Francisco, province of Azuay, Ecuador (S3.30829, W79.47079; 862 m). QCAZ 14446, adult male collected by Fernando Ayala, Steven Poe and Chris Anderson on January 10, 2016 at Ponce Enríquez–El Coca, province of Azuay, Ecuador (S3.03197, W79.64615; 1206 m). ZSFQ D503, adult male collected by Diego Cisneros-Heredia on June 07, 2000 at Cerro La Mocora, Parque Nacional Machalilla, province of Manabí, Ecuador (S1.60379, W80.70191; 818 m).

##### Diagnosis.

*Sibon bevridgelyi* is placed in the genus *Sibon* based on phylogenetic evidence (Fig. 3) and on having the labial beneath primary temporal conspicuosly higher than other labials. The species differs from all described species of *Sibon* based on the following combination of characters: (1) 15/15/15 smooth dorsals with enlarged vertebral row (1.3–1.7 times as wide as adjacent rows); (2) seven supralabials with 4^th^ and 5^th^ contacting orbit or eight supralabials with 5^th^ and 6^th^ contacting orbit; (3) one pair of infralabials in contact behind symphysial; (4) postmental absent; (5) 175–193 ventrals in males, 193 in the single female; (6) 80–94 divided subcaudals in males, 98 in the single female; (7) dorsal and ventral ground color pale yellow with or without irregular black bands, and with a black stippled disruptive pattern of irregular rusty to reddish brown blotches that are separated from each other by light interspaces (Figs 6, 2b, c); bands incomplete and stippling not prominent or absent on ventral surfaces; head heavily speckled or blotched with black or rusty pigment; eyes light slate blue to pale goldenrod with black speckles and reticulations; (8) 349–732 mm SVL in males, 786 mm in the single female; (9) 124–268 mm TL in males, 204 mm in the single female.

##### Comparisons.

*Sibon bevridgelyi* is most similar to *S. nebulatus*, from which it differs on the basis of its distinctive coloration (Figs 6, 2b, c). In *S. nebulatus* (Figs 2e, f), the dorsal and ventral color is a combination of mainly black to dark-brown blotches or bands on a gray to grayish brown background (interblotch) color; the dorso-lateral blotches can partly be bordered by white to rosy scales or edges. In some regions, the blackish pattern and gray ground color is often replaced by dark and light brown tones (e.g., in Venezuela, adjacent regions in Colombia, and Trinidad and Tobago); the spaces between the blotches are heavily invaded by blotch color and strongly stippled, spotted and mottled with white and black pigment. Although *S. bevridgelyi* also has a disruptive pattern, the diagnostic white and gray pigment of *S. nebulatus* from Central America and northern South America is lacking in *S. bevridgelyi*. Instead of white pigment, there is golden yellow; instead of gray, the dominant ground color is bright rusty brown to maroon. Additionally, the infralabials and the whitish throat in *S. nebulatus* from Central America and northern South America are heavily stippled or at least partly interrupted with black pigment, whereas in *S. bevridgelyi* the infralabials and the throat are immaculate or have few scattered blotches (Fig. 7b). Finally, the black blotches and stippling diagnostic of *S. nebulatus* are lacking in the majority of the specimens of *S. bevridgelyi*. Specimens of *S. nebulatus* with rosy gray or reddish brown ground color have white (instead of yellowish) blotches and stippling. Genetic divergence in a 521 bp long fragment of the mitochondrial Cytb gene between *S. bevridgelyi* and *S. nebulatus leucomelas* is 1.9–2.5%, whereas intraspecific distances are less than 0.4% in both species.

##### Description of holotype.

Adult male, SVL 602 mm, tail length 186 mm (31% SVL); head length 20.9 mm (3% SVL) from tip of snout to commissure of mouth; head width 12.4 mm (59% head length) taken at broadest point; snout-orbit distance 21 mm; head distinct from neck; snout short, blunt in dorsal and lateral outline; rostral 3.5 mm wide, broader than high; internasals 1.9 mm wide, broader than long; prefrontals 4.4 mm wide, longer than broad, entering orbit; supraocular 4.4 mm long, longer than broad; frontal 4.1 mm long, pentagonal and rounded, in contact with prefrontals, supraoculars, and parietals; parietals 7.7 mm long, longer than broad; nasal weakly divided, in contact with first three supralabials, loreal, prefrontal, internasal, and rostral; loreal 3.7 mm long, longer than high, entering the orbit; eye diameter 3.9 mm; pupil semi-elliptical; no preocular; two postoculars; temporals 1+3 on the right side, 2+3 on the left side; eight supralabials with 5^th^ and 6^th^ contacting orbit on the right side, seven supralabials with 4^th^ and 5^th^ contacting orbit on the left side; symphysial separated from chinshields by the first pair of infralabials; nine infralabials, 1–7 contacting chinshields; anterior pair of chinshields broader than long, posterior pair longer than broad; dorsal scales in 15/15/15 rows, smooth, without apical pits; 184 ventrals; 80 divided subcaudals; cloacal plate single.

##### Natural history.

Specimens of *Sibon bevridgelyi* have been found active at night (20h56–03h56) on arboreal vegetation 30–500 cm above the ground in secondary and primary semideciduous foothill forest, pastures, and cacao plantations, usually close to streams. QCAZ 14444 was found feeding on a snail. In captivity, MZUA.RE.0142 fed on slugs and snails. By daytime, one individual (not collected) was found hidden under tree bark, and another (ZSFQ D503) was found coiled on the center of a palm tree about 2 m above the ground. DHMECN 9483 was collected in sympatry with *Dipsas andiana* and *D. bobridgelyi* at Reserva Biológica Buenaventura.

##### Distribution.

Northwestern Peru in the department of Piura, and southwestern Ecuador in the provinces of Azuay, Chimborazo, El Oro, Guayas, Los Ríos and Manabí at elevations between 5 and 1206 m (Fig. 8).

##### Etymology.

The specific epithet honors the late Prof. Beverly S. Ridgely, life-long birder and conservationist, and father of Robert S. Ridgely, well known in Ecuadorian ornithological circles and co-author of *The Birds of Ecuador*. Though he never got to visit Buenaventura, from afar Bev continued to delight in the conservation successes of Fundación Jocotoco, which now owns and manages one of the few protected areas where the Vulnerable *Sibon bevridgelyi* is known to occur.

##### Conservation status.

We consider *Sibon bevridgelyi* to be Vulnerable following B2a,b(i,iii) IUCN criteria (IUCN 2001) because its area of occupancy is estimated to be less than 2,000 km^2^, it is known only from 15 patches of forest lacking connectivity between them, and its habitat is severely fragmented and declining in extent and quality due to deforestation. Furthermore, only three of the localities (Parque Nacional Machalilla, Reserva Buenaventura, and Reserva Ayampe) where *S. bevridgelyi* occurs are currently protected.

#### Dipsas bobridgelyi sp. n.

Taxon classificationAnimaliaSquamataDipsadidae

http://zoobank.org/6B9E1F98-77A9-41F7-8CF1-F56404F8CBD0

Figs 1c , 9 , 10

##### Proposed standard English name.

Bob Ridgely’s Snail-Eater

##### Proposed standard Spanish name.

Caracolera de Bob Ridgely

##### Holotype.

MZUTI 5417 (Figs 9, 10), adult male collected by Matthijs Hollanders on August 01, 2017 at Reserva Buenaventura, province of El Oro, Ecuador (S3.65467, W77.76794; 524 m).

##### Paratypes.

DHMECN 11527, adult female collected by Juan Carlos Sánchez-Nivicela, Karem López, Verónica Urgilés, Bruno Timbe, Elvis Celi and Valentina Posse at Remolino, province of El Oro, Ecuador (S3.56551, W79.91948; 229 m). MZUTI 3266, adult female collected by Lucas Bustamante on October 06, 2013. MZUTI 5414, adult male collected by Matthijs Hollanders and Paulina Romero on June 08, 2017. QCAZ 1706, adult male collected by Fernando Ayala, Steven Poe, and Chris Anderson on March 03, 1994 at Ponce Enríquez, province of Azuay, Ecuador (S3.06547, W79.74358; 39 m).

##### Diagnosis.

*Dipsas bobridgelyi* is placed in the genus *Dipsas* based on phylogenetic evidence (Fig. 3), and the absence of a labial that is noticeably higher than other labials and in contact with the postocular, primary, and secondary temporals. The species differs from all described species of *Dipsas* based on the following combination of characters: (1) 15/15/15 smooth dorsals with enlarged vertebral row (2.1–2.2 times as wide as adjacent rows); (2) loreal and prefrontal in contact with orbit; (3) 9 supralabials with 4^th^ and 5^th^ contacting orbit; (4) one pair of infralabials in contact behind symphysial; (5) 180–201 ventrals in males, 178–184 in females; (6) 95–117 divided subcaudals in males, 96–98 in females; (7) dorsal and ventral color made up of 30–35 bold black body rings (up to 7–12 vertebral scales long) separated from each other by narrow (up to 3–4 vertebral scales long) dingy white interspaces; dorsal aspect of interspaces heavily speckled with rusty and black pigment; ventral surfaces lacking speckling; ground color of head dingy white with various degrees of scattered black pigment that coalesce on the top of the head, and various degrees of rusty speckling concentrated on the snout, nape and sides of the head; iris rich dark brown; (8) 372–478 mm SVL in males, 286–404 mm in females; (9) 158–212 mm TL in males, 117–158 mm in females.

##### Comparisons.

*Dipsas bobridgelyi* is most similar to *D. gracilis*, from which it differs in coloration. In *D. gracilis* (Figs 1h, i), the black rings are up to 10–16 vertebral scales long and the interspaces are up to 5–7 scales long, whereas in *D. bobridgelyi* the black rings and interspaces are shorter, up to 8–9 and 3–4 vertebral scales long, respectively. In *D. gracilis*, the head plates are either completely black or black scattered with reddish brown, whereas in *D. bobridgelyi* the head plates are heavily stippled with white and tan pigment, especially on the prefrontals and internasals. In all known specimens of *D. bobridgelyi*, the ground color of the interspaces is white with contrasting reddish-tan pigment in the center, whereas in *D. gracilis* the ground color of the light interspaces on body and tail is either completely light brown or light reddish white, gradually becoming reddish brown towards the center. Finally, the nape and temporal region of the head in *D. gracilis* are either immaculate light reddish brown or marked with bold black speckles, whereas in *D. bobridgelyi* they are an irregular mix of fine speckling of white, rusty, and black pigments. Genetic divergence in a 689 bp long fragment of the mitochondrial Cytb gene between *D. bobridgelyi* and *D. gracilis* is 8.7–9.0%, whereas intraspecific distances are less than 0.3% in both species.

##### Description of holotype.

Adult male, SVL 372 mm, tail length 158 mm (43% SVL); head length 15.1 mm (4% SVL) from tip of snout to commissure of mouth; head width 8.1 mm (54% head length) taken at broadest point; snout-orbit distance 4.3 mm; head distinct from neck; snout short, blunt in dorsal and lateral outline; rostral 2.4 mm wide, broader than high; internasals 2.3 mm wide, broader than long; prefrontals 2.5 mm wide, longer than broad and contacting orbit; supraocular 3.2 mm long, longer than broad; frontal 3.9 mm long, hexagonal, in contact with prefrontals, supraoculars, and parietals; parietals 4.7 mm long, longer than broad; nasal divided, in contact with first three supralabials, loreal, prefrontal, internasal, and rostral; loreal 1.8 mm long, slightly higher than long, entering the orbit; eye diameter 2.7 mm; pupil semi-elliptical; no preocular; two postoculars; temporals 2+3; nine supralabials, 4^th^ and 5^th^ contacting orbit; symphysial separated from chinshields by the first pair of infralabials; 13 infralabials, 1–7 contacting chinshields; anterior pair of chinshields longer than broad, posterior pair broader than long; dorsal scales in 15/15/15 rows, smooth, without apical pits; 182 ventrals; 101 divided subcaudals; cloacal plate single.

##### Natural history.

Individuals of *Dipsas bobridgelyi* have been found active at night (19h00–23h26) on arboreal vegetation 100–250 cm above the ground in secondary semi-deciduous foothill forest. MZUTI 5414 was found feeding on a snail.

##### Distribution.

Foothills of the southwestern Ecuadorian Andes in the provinces of Azuay and El Oro, and northwestern Peruvian Andes in the department of Tumbes, at elevations between 39 and 572 m (Fig. 4).

##### Etymology.

This species is named in honor of Dr. Robert “Bob” S. Ridgely, a leading ornithologist and distinguished conservationist who has dedicated almost 50 years of his life to the study and conservation of birds and biodiversity across Latin America. Bob is the President of Rainforest Trust and for the past twenty years has been a major driver of conservation in Ecuador through Fundación Jocotoco, which he helped establish twenty years ago. In 1980, Bob visited the type locality of *Dipsas bobridgelyi* (Buenaventura, meaning "good fortune"), now known to be a key area for the conservation of biodiversity. Bob embarked on conservation and worked diligently to raise funds through Rainforest Trust for the past 18 years to purchase private properties and establish what is now the Reserva Buenaventura of Fundación Jocotoco.

##### Conservation status.

We consider *Dipsas bobridgelyi* to be Endangered following the IUCN criteria B1a,b(i,iii) (IUCN 2001) because its extent of occurrence is estimated to be less than 5,000 km^2^, it is known only from 4 patches of forest lacking connectivity between them, and its habitat is severely fragmented and declining in extent and quality due to deforestation. Furthermore, only two of the localities (Buenaventura reserve and Reserva Nacional de Tumbes) where *D. bobridgelyi* occurs are currently protected.

##### Remarks.

Cadle (2005) and Harvey (2008) examined MUSM 17589 from Tumbes department, Peru, and concluded that it was *Dipsas gracilis*. Although we did not examine this specimen, we believe that it corresponds to *D. bobridgelyi* based on Cadle’s (2005) color description (i.e., head white with many irregular black markings on the top and sides).

#### Dipsas georgejetti sp. n.

Taxon classificationAnimaliaSquamataDipsadidae

http://zoobank.org/AAE7F2F6-8082-4FEA-AE59-BC0901FE9211

Figs 11 , 12

##### Proposed standard English name.

George Jett’s Snail-Eater

##### Proposed standard Spanish name.

Caracolera de George Jett

##### Holotype.

MZUTI 5411 (Figs 11, 12), adult male collected by Melissa Costales on August 31, 2017 at Cabuyal, province of Manabí, Ecuador (S0.19698, W80.29059; 15 m).

##### Paratypes.

DHMECN 11639, adult male collected by Jacinto Bravo in 2014 at Montecristi, province of Manabí, Ecuador (S1.04694, W80.65766; 136 m). DHMECN 11646, adult male collected by Félix Almeida in 2014 at Rocafuerte, province of Manabí, Ecuador (S0.92371, W80.45212; 19 m). MZUA.RE.0121 and MZUA.RE.0122, adult female and adult male, respectively, collected by Juan Carlos Sánchez-Nivicela at El Aromo, province of Manabí, Ecuador (S1.04665, W80.83227; 295 m). QCAZ 10589, adult male collected at El Aromo, province of Manabí, Ecuador (S1.04665, W80.83227; 295 m). QCAZ 9125, adult male collected at Cerro Blanco, province of Guayas, Ecuador (S2.17465, W80.02135; 147 m). USNM 142595, juvenile of undetermined sex collected on December 1959 at 10 mi N of Guayaquil, province of Guayas (S1.96418, W79.87988; 5 m). ZSFQ D606, juvenile male collected by Diego F. Cisneros-Heredia at the foothills of Cerro La Mocora, Parque Nacional Machalilla, province of Manabí, Ecuador (S1.59817, W80.75431; 308 m).

##### Diagnosis.

*Dipsas georgejetti* is placed in the genus *Dipsas* based on phylogenetic evidence (Fig. 3) and the absence of a labial that is noticeably higher than other labials and in contact with the postocular, primary and secondary temporals. The species differs from all described species of *Dipsas* based on the following combination of characters: (1) 15/15/15 smooth dorsals with a slightly enlarged vertebral row (1–1.4 times as wide as adjacent rows); (2) loreal and prefrontal in contact with orbit; (3) 7 supralabials with 4^th^ and 5^th^ (3^th^–5^th^ in DHMECN 11646) contacting orbit; (4) no infralabials in contact behind symphysial; (5) 172–180 ventrals in males, 177 in one female; (6) 69–86 divided subcaudals in males, 58 in one female; (7) dorsal ground color light sandy brown with a pattern of 53–61 drab to brown black-edged middorsal blotches that are wider (6–7 vertebral scales long) and solid down to the edges of the ventrals on the first one third of the body, but becoming narrower (1–3 vertebral scales long) and broken up laterally towards the tail; interspaces finely speckled with brown pigment; ground color of the head light sandy brown with bold dark brown to black irregular blotches scattered on head plates and edging supralabials; ventral surfaces sandy brown with fine black speckling; iris sandy brown with dense dark brown speckling; (8) 270–711 mm SVL in males, 856 mm in one female; (9) 87–170 mm TL in males, 150 mm in one female.

##### Comparisons.

*Dipsas georgejetti* is most similar to *D. oswaldobaezi*, *D. williamsi*, *D. oligozonata*, and *D. vagrans*, in that order, all of which were previously included in the genus *Sibynomorphus*. From *D. oswaldobaezi* (Figs 13, 14) and *D. williamsi*, it differs in having 7 supralabials with 4^th^ and 5^th^ bordering the eye (instead of 6 with 3^rd^ and 4^th^ bordering the eye). It further differs from *D. williamsi* in having the first supralabial not in contact with prefrontal (vs. in broad contact in *D. williamsi*). From *D. oligozonata* (Fig. 1o) and *D. vagrans*, it differs in having more than 160 ventrals. *Dipsas georgejetti* further differs from *D. oligozonata* in having distinct bold crossbands at least middorsally along the whole length of the body, instead of being present only on the anterior half of the body. Genetic divergence in a 529 bp long fragment of the mitochondrial Cytb gene between *D. georgejetti* and *D. oswaldobaezi* is 8.3%, whereas intraspecific distances are less than 0.4% in *D. georgejetti*. For the same fragment, the distance between *D. georgejetti* and *D. williamsi* is 7.8–7.9%.

##### Description of holotype.

Adult male, SVL 315 mm, TL 87 mm (28% SVL); head length 13.6 mm (4% SVL) from tip of snout to commissure of mouth; head width 8.4 mm (62% head length) taken at broadest point; snout-orbit distance 3.5 mm; head distinct from neck; snout short, blunt in dorsal and lateral outline; rostral 2.0 mm wide, broader than high; internasals 1.7 mm wide, broader than long; prefrontals 2.5 mm wide, longer than broad and contacting orbit; supraocular 3.4 mm long, longer than broad; frontal 3.3 mm long, pentagonal, in contact with prefrontals, supraoculars, and parietals; parietals 5.5 mm long, longer than broad; nasal divided, in contact with first two supralabials, loreal, prefrontal, internasal, and rostral; loreal 1.7 mm long, slightly higher than long, entering orbit; eye diameter 2.8 mm; pupil semi-elliptical; no preocular; two postoculars; temporals 2+2; seven supralabials, 4^th^ and 5^th^ contacting orbit; symphysial in contact with first pair of chinshields; nine infralabials, 1–6 contacting chinshields; anterior pair of chinshields longer than broad, posterior pair broader than long; dorsal scales in 15/15/15 rows, smooth, without apical pits; 178 ventrals; 69 divided subcaudals; cloacal plate single.

##### Natural history.

The holotype was active during a dry night after a sunny day. It was perched on tangled vegetation 130 cm above the ground in dry shrubland besides recently cleared pasture. MZUA.RE0121 and MZUA.RE0122 were found actively moving at night between the branches 80–200 cm above the ground. ZSFQ D606 was found active during daytime after bulldozers opened a track in old-growth forest.

##### Distribution.

**Figure 5.**
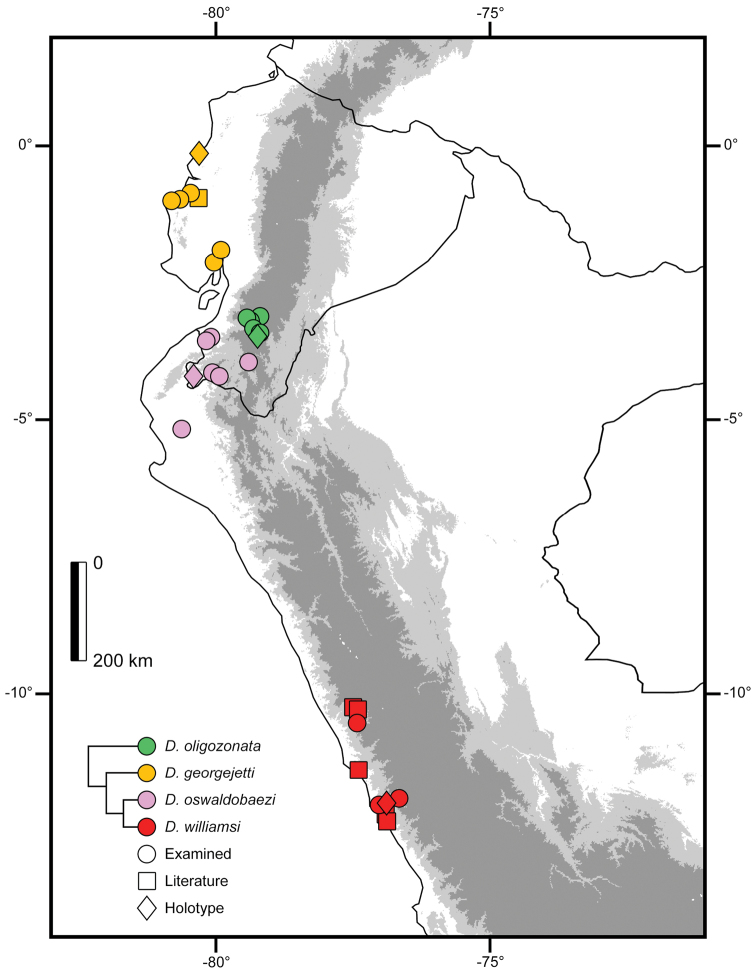
Distribution of *Dipsas georgejetti*, *D. oligozonata*, *D. oswaldobaezi*, and *D. williamsi* in Ecuador and Peru. Figures represent known localities.

Deciduous and semideciduous forests along the central Pacific coast in Ecuador in the provinces of Manabí and Guayas, at elevations between 5 and 317 m (Fig. 5).

##### Etymology.

The specific name *georgejetti* honors George Jett, who has been a long-time donor to Rainforest Trust and has supported the reserves of Fundación Jocotoco in Ecuador. He is an international traveler with a passion for reptiles, amphibians, and birds.

##### Conservation status.

We consider *Dipsas georgejetti* to be Vulnerable following the IUCN criteria A1c,B1a,b(iii, iv) (IUCN 2001) because its extent of occurrence is estimated to be 10,193 km^2^, it is known only from 9 localities effectively corresponding to 4 patches of forest lacking connectivity between them, and its habitat is severely fragmented and declining in extent and quality due to deforestation. At the type locality, *D. georgejetti* was found in a patch of deciduous forest of 13 km^2^ that was being cleared to accommodate cattle pastures. One of the localities, 15 km N of Guayaquil, where *D. georgejetti* was collected in 1959, is now completely deforested, which suggests that this arboreal species is no longer present there.

**Figure 6.**
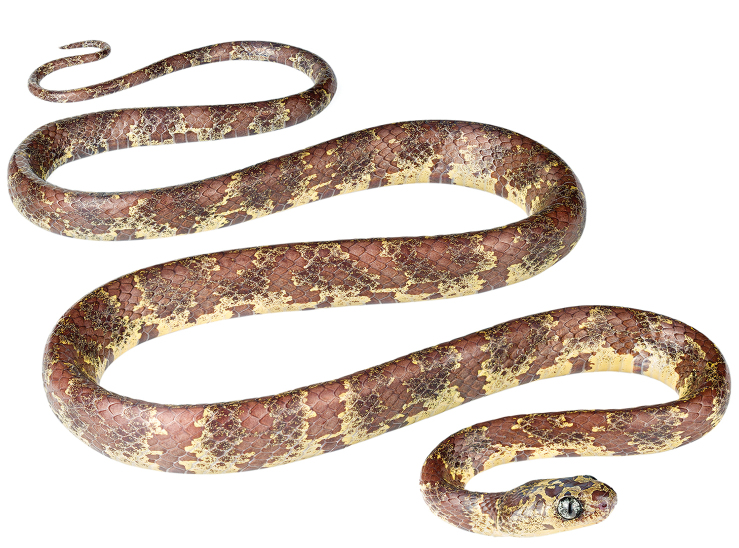
Adult male holotype of *Sibon bevridgelyi*. MZUTI 5416.

#### Dipsas oswaldobaezi sp. n.

Taxon classificationAnimaliaSquamataDipsadidae

http://zoobank.org/EA450E16-23F3-4A84-B067-00614621FFD1

Figs 13 , 14

Sibynomorphus oligozonatus Cadle, 2007: 195 (part).

##### Proposed standard English name.

Oswaldo Báez’ Snail-Eater

##### Proposed standard Spanish name.

Caracolera de Oswaldo Báez

##### Holotype.

QCAZ 10369 (Fig. 13), adult female collected by Silvia Aldás and Gabriel Zapata on March 03, 2010 at Quebrada El Faique, province of Loja, Ecuador (S4.17889, W80.04226; 1004 m).

##### Paratypes.

BMNH1935.11.3.108, adult female collected by Clodoveo Carrión in the valley of Catamayo, province of Loja, Ecuador (S3.98064, W79.35928; 1289 m). MUSM 2192, adult male collected by Otavio Ruíz in Piura (department or city not specified), Peru. MZUA.RE.0286, adult of undetermined sex collected by Valentina Posse on December 2015 at Huaquillas, province of El Oro, Ecuador (S3.54115, W80.08646; 39 m). QCAZ 14051, adult of undetermined sex collected by Paul Székely and Diana Székely on March 18, 2015 at Reserva Ecológica Arenillas, province of El Oro, Ecuador (S3.62110, W80.17513; 41 m). QCAZ 14060, adult of undetermined sex collected by Paul Székely and Diana Székely on June 16, 2015 at Guabillo, province of El Oro, Ecuador (S3.60346, W80.18139; 44 m). QCAZ 15108, adult female collected by Diego Almeida, Darwin Núñez, Eloy Nusirquia, Santiago Guamán and Guadalupe Calle on November 12, 2016 at Reserva La Ceiba-Pilares, province of Loja, Ecuador (S4.27502, W80.32805; 534 m) (Fig. 14).

##### Diagnosis.

*Dipsas oswaldobaezi* is placed in the genus *Dipsas* based on phylogenetic evidence (Fig. 3) and the absence of a labial that is noticeably higher than other labials and in contact with the postocular, primary and secondary temporals. The species differs from all described species of *Dipsas* based on the following combination of characters: (1) 15/15/15 smooth dorsals with a slightly enlarged vertebral row (1–1.2 times as wide as adjacent rows); (2) loreal and prefrontal in contact with orbit; (3) six supralabials with 3^rd^ and 4^th^ contacting orbit; (4) no infralabials in contact behind symphysial; (5) 163–179 ventrals in males, 177–179 in females; (6) 68–70 divided subcaudals in males, 65–66 in females; (7) dorsal ground color light sandy brown with a pattern of 55–63 drab to brown black-edged middorsal blotches that are wider (7–9 vertebral scale rows) and solid down to the edges of the ventrals on the first one third of the body, but becoming narrower (1–3 vertebral scales long) and broken up laterally towards the tail; interspaces finely speckled with brown pigment; ground color of the head light sandy brown with a thin light cream nuchal collar and bold dark brown to black irregular blotches scattered on head plates and edging supralabials; ventral surfaces sandy brown with fine black speckling (Fig. 13b); iris sandy brown with dense dark brown speckling; (8) 277–348 mm SVL in males, 407–428 mm in females; (9) 85–114 mm TL in males, 110–122 mm in females.

##### Comparisons.

*Dipsas oswaldobaezi* is most similar to *D. williamsi*, *D. georgejetti*, *D. oligozonata*, and *D. vagrans*, in that order, all of which were previously included in the genus *Sibynomorphus*. From *D. williamsi*, it differs in having 7–9 infralabials (vs. 10 in *D. williamsi*), first supralabial not in contact with prefrontal (vs. in broad contact in *D. williamsi*), and dorsal blotches that are lighter in the middle (vs. dark solid blotches). From *D. georgejetti* (Figs 11, 12), it differs in having 6 supralabials with 3^rd^ and 4^th^ bordering the eye (vs. 7 supralabials with 4^th^ and 5^th^ bordering the eye in *D. georgejetti*). From *D. oligozonata* (Fig. 1o) and *D. vagrans*, it differs in having more than 160 ventrals. *Dipsas oswaldobaezi* further differs from *D. oligozonata* in having distinct bold crossbands at least middorsally along the whole length of the body, instead of being present only on the anterior half of the body. Genetic divergence in a 529 bp long fragment of the mitochondrial Cytb gene between *D. oswaldobaezi* and *D. williamsi* is 4.0–4.2%, whereas intraspecific distances are less than 0.2% in *D. williamsi*. For the same fragment, the distance between *D. oswaldobaezi* and *D. georgejetti* is 8.3%.

##### Description of holotype.

Adult female, SVL 277 mm, tail length 85 mm (31% SVL); head length 9.5 mm (3.4% SVL) from tip of snout to commissure of mouth; head width 7.3 mm (76% head length) taken at broadest point; snout-orbit distance 3.3 mm; head distinct from neck; snout short, blunt in dorsal and lateral outline; rostral 2.1 mm wide, broader than high; internasals 1.2 mm wide, broader than long; prefrontals 2.2 mm wide, slightly broader than long and contacting orbit; supraocular 2.6 mm long, longer than broad; frontal 2.9 mm long, pentagonal, in contact with prefrontals, supraoculars, and parietals; parietals 4.2 mm long, longer than broad; nasal not divided, in contact with first supralabial, loreal, prefrontal, internasal, and rostral; loreal 1.3 mm long, longer than high, entering orbit; eye diameter 2.2 mm; pupil semi-elliptical; no preocular; two postoculars; temporals 2+2; 6 supralabials, 3^rd^ and 4^th^ contacting orbit; symphysial separated from chinshields by the first pair of infralabials; 9/8 (right/left) infralabials, 1–6/1–5 contacting chinshields; both pairs of chinshields longer than broad; dorsal scales in 15/15/15 rows, smooth, without apical pits; 179 ventrals; 70 divided subcaudals; cloacal plate single.

##### Natural history.

Individuals of *Dipsas oswaldobaezi* have been found active by night on vegetation or at ground level in forested environments, pastures, or rural gardens. One individual (QCAZ 15108) was found hidden under leaf litter during daytime. Two individuals (MZUA.RE.0286 and QCAZ 14060) were found dead on roads.

##### Distribution.

Deciduous and semideciduous lowland to lower montane forests and dry lowland shrublands in southwestern Ecuador (provinces of Loja and El Oro) and northwestern Peru (department of Tumbes), at elevation between 39 and 1289 m (Fig. 5).

##### Etymology.

The specific name *oswaldobaezi* honors Dr. Oswaldo Báez, a renowned Ecuadorian biologist and researcher who has dedicated his life to the teaching of science, scientific thinking, and the conservation of nature. Oswaldo Báez has played a major role in science education in Ecuador through many popular science articles and books.

##### Conservation status.

We consider *Dipsas oswaldobaezi* to be Vulnerable following the IUCN criteria B1a,b(iii, iv) (IUCN 2001) because its extent of occurrence is estimated to be 8,605 km^2^; it is known only from eight localities effectively corresponding to four patches of forest lacking connectivity between them, and its habitat is severely fragmented and declining in extent and quality due to deforestation.

##### Remarks.

In his revision of *Dipsas oligozonata*, Cadle (2007) allocated three additional specimens (AMNH 110587, BMNH 1935.11.3.108 and MUSM 2192) to a species known only from the holotype (EPN 3612), collected at Zhila, province of Azuay (S3.50280, W79.18808; 2795 m) (Fig. 5). AMNH 110587 was collected ca. 34 km airline distance from the type locality at an elevation of 2204 m, and it resembles the holotype in both color and lepidosis. However, BMNH 1935.11.3.108 and MUSM 2192 have more than 160 ventral scales and have broad dark brown crossbars that are at least twice as long as those present in both the holotype, AMNH 110587 and in the other four specimens of *D. oligozonata* examined by us (Table 2; Fig. 1o), all of which have fewer than 160 ventral scales and come from elevations between 2102 and 2891 m in the watershed of the Río Jubones (Fig. 5). The coloration and ventral scale counts in BMNH 1935.11.3.108 and MUSM 2192 are more similar to *D. oswaldobaezi*, and we designated them as paratypes of this species.

**Figure 7.**
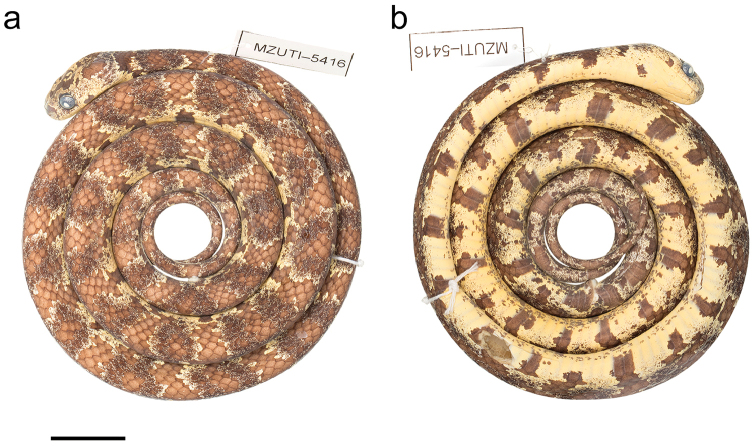
Adult male holotype of *Sibon bevridgelyi* MZUTI 5416 in (a) dorsal and (b) ventral view. Scale bar: 1 cm.

**Figure 8.**
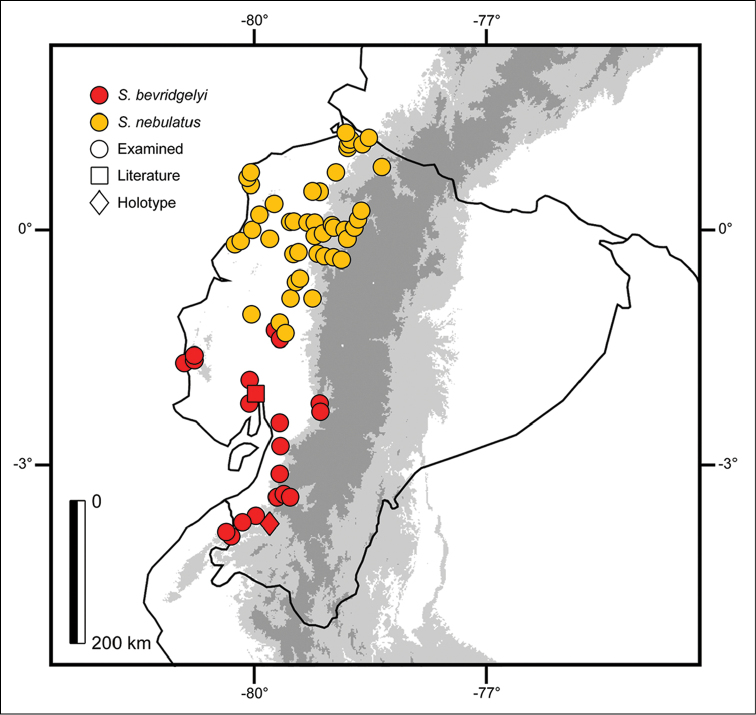
Distribution of *Sibon nebulatus* and *S. bevridgelyi* in Ecuador. Figures represent known localities.

**Table 2.** Locality data for specimens examined in this study. Coordinates represent actual GPS readings taken at the locality of collection or georeferencing attempts from gazetteers under standard guidelines, though some variation from the exact collecting locality will be present. Similarly, elevations are taken from Google Earth, and may not exactly match the elevations as originally reported. Specimens listed here but not under Appendix 3 were examined indirectly (e.g., through photographs).

Species	Voucher	Country	Province	Locality	Latitude	Longitude	Elev. (m)
*D. andiana*	MZUA.RE.0230	Ecuador	Cañar	Ocaña	-2.48807, -79.18758		923
*D. andiana*	MHNG 2250.053	Ecuador	Cotopaxi	Las Pampas	-0.43021, -78.96663		1590
*D. andiana*	MZUTI 5413	Ecuador	El Oro	Reserva Buenaventura	-3.65477, -79.76830		497
*D. andiana*	MZUTI 3501	Ecuador	Pichincha	Mashpi lodge	0.16567, -78.88656		860
*D. andiana*	MZUTI 3505	Ecuador	Pichincha	Valle Hermoso–Los Bancos	-0.01371, -79.09462		571
*D. andiana*	ZSFQ D116	Ecuador	Pichincha	Tandayapa	0.00205, -78.67880		1734
*D. andiana*	ZSFQ D117	Ecuador	Pichincha	Hacienda La Joya	0.08291, -78.98311		763
*D. andiana*	ZSFQ D115	Ecuador	Manabí	5km W Puerto López	-1.59045, -80.84087		7
*D. bobridgelyi*	QCAZ 1706	Ecuador	Azuay	Ponce Enríquez	-3.06547, -79.74358		39
*D. bobridgelyi*	DHMECN 11527	Ecuador	El Oro	Remolino	-3.56551, -79.91948		229
*D. bobridgelyi*	MZUTI 3266	Ecuador	El Oro	Reserva Buenaventura	-3.65467, -79.76794		524
*D. bobridgelyi*	MZUTI 5414	Ecuador	El Oro	Reserva Buenaventura	-3.65310, -79.76336		572
*D. bobridgelyi*	MZUTI 5417	Ecuador	El Oro	Reserva Buenaventura	-3.65467, -79.76794		524
*D. catesbyi*	MHNG 2220.054	Ecuador	Morona Santiago	Macas	-2.31670, -78.11670		972
*D. catesbyi*	MHNG 2238.005	Ecuador	Morona Santiago	San Pablo de Kantesiya	-0.25001, -76.41849		250
*D. catesbyi*	USNM 283949	Ecuador	Morona Santiago	Sucúa	-2.45663, -78.16784		829
*D. catesbyi*	DHMECN 11555	Ecuador	Napo	El Reventador	-0.04669, -77.52898		1428
*D. catesbyi*	QCAZ 181	Ecuador	Napo	Hollín–Loreto	-0.74087, -77.51945		1020
*D. catesbyi*	MHNG 2220.052	Ecuador	Napo	San Rafael	-0.10354, -77.58337		1246
*D. catesbyi*	QCAZ 210	Ecuador	Napo	San Rafael	-0.09669, -77.58995		1464
*D. catesbyi*	MHNG 2206.086	Ecuador	Orellana	Hacienda Primavera	-0.48689, -76.63581		267
*D. catesbyi*	MHNG 2435.097	Ecuador	Pastaza	Puyo	-1.46678, -77.98335		953
*D. catesbyi*	QCAZ 5108	Ecuador	Pastaza	Villano B	-1.49961, -77.48234		341
*D. catesbyi*	MHNG 2249.001	Ecuador	Sucumbíos	El Reventador	-0.04480, -77.52858		1476
*D. catesbyi*	QCAZ 28	Ecuador	Sucumbíos	El Reventador	-0.04669, -77.52898		1428
*D. catesbyi*	MHNG 2238.014	Ecuador	–	–	–	–	–
*D. catesbyi*	MHNG 2307.091	Ecuador	–	–	–	–	–
*D. catesbyi*	MZUTI 4736	Ecuador	–	–	–	–	–
*D. catesbyi*	MZUTI 4999	Ecuador	–	–	–	–	–
*D. elegans*	MHNG 2435.084	Ecuador	Cotopaxi	Cutzualo	-0.54497, -78.91891		1952
*D. elegans*	MHNG 2440.098	Ecuador	Cotopaxi	Galápagos	-0.40583, -78.96667		1781
*D. elegans*	DHMECN 1693	Ecuador	Cotopaxi	Hacienda “La Mariela”	-1.14757, -79.09126		1256
*D. elegans*	MHNG 2457.078	Ecuador	Cotopaxi	Las Damas	-0.38402, -78.96741		1678
*D. elegans*	MHNG 2249.019	Ecuador	Cotopaxi	Las Pampas	-0.43021, -78.96663		1590
*D. elegans*	MHNG 2413.074	Ecuador	Cotopaxi	Palo Quemado	-0.61962, -78.99066		2402
*D. elegans*	USNM 285957	Ecuador	Pichincha	2.9 km SW of Tandayapa	0.00578, -78.67867		1844
*D. elegans*	MHNG 2399.072	Ecuador	Pichincha	Ilaló	-0.26166, -78.44444		2579
*D. elegans*	MZUTI 3695	Ecuador	Pichincha	Tambotanda	-0.02011, -78.65101		1875
*D. elegans*	MZUTI 3317	Ecuador	Pichincha	Tandapi	-0.42278, -78.79611		1550
*D. elegans*	MHNG 2457.079	Ecuador	Santo Domingo	Chiriboga	-0.22841, -78.76725		1813
*D. elegans*	MHNG 2308.002	Ecuador	Santo Domingo	Hacienda Las Palmeras	-0.24520, -78.84806		1876
*D. elegans*	MHNG 2220.093	Ecuador	–	–	–	–	–
*D. elegans*	MZUTI 3316	Ecuador	–	–	–	–	–
*D. ellipsifera*	MZUTI 4931	Ecuador	Carchi	Chilma Bajo	0.86274, -78.05080		2071
*D. ellipsifera*	QCAZ 14855	Ecuador	Carchi	Quebrada Golondrinas	0.83210, -78.12324		1737
*D. ellipsifera*	QCAZ 15225	Ecuador	Carchi	Río Pailón	0.95643, -78.23448		1669
*D. ellipsifera*	MHNG 2220.048	Ecuador	Imbabura	Cotacachi	0.29395, -78.26682		2446
*D. gracilis*	QCAZ 4137	Ecuador	Cañar	Manta Real	-2.55367, -79.36425		257
*D. gracilis*	QCAZ 3504	Ecuador	Esmeraldas	Angostura	1.02164, -78.86295		31
*D. gracilis*	QCAZ 10549	Ecuador	Esmeraldas	Caimito	0.69546, -80.08990		118
*D. gracilis*	QCAZ 14495	Ecuador	Esmeraldas	Estero Gasparito	0.91296, -78.84066		80
*D. gracilis*	QCAZ 2629	Ecuador	Esmeraldas	Fauna Granja Tropical	0.66152, -79.53875		29
*D. gracilis*	QCAZ 7321	Ecuador	Esmeraldas	La Mayronga	1.04361, -79.27786		14
*D. gracilis*	QCAZ 13738	Ecuador	Esmeraldas	Tundaloma	1.18166, -78.74945		74
*D. gracilis*	MZUA.RE.0280	Ecuador	Guayas	Naranjal	-2.72302, -79.63172		58
*D. gracilis*	MZUA.RE.0281	Ecuador	Guayas	Naranjal	-2.72302, -79.63172		58
*D. gracilis*	QCAZ 12478	Ecuador	Guayas	Río Patul	-2.55548, -79.37180		266
*D. gracilis*	QCAZ 8432	Ecuador	Los Ríos	Buena Fe	-0.89306, -79.48957		104
*D. gracilis*	MHNG 2309.038	Ecuador	Los Ríos	Río Palenque	-0.58333, -79.36667		173
*D. gracilis*	QCAZ 10196	Ecuador	Los Ríos	Río Palenque	-0.58333, -79.36667		173
*D. gracilis*	USNM 285477	Ecuador	Los Ríos	Río Palenque	-0.58333, -79.36667		173
*D. gracilis*	USNM 285478	Ecuador	Los Ríos	Río Palenque	-0.58333, -79.36667		173
*D. gracilis*	USNM 285479	Ecuador	Los Ríos	Río Palenque	-0.58333, -79.36667		173
*D. gracilis*	USNM 285480	Ecuador	Los Ríos	Río Palenque	-0.58333, -79.36667		173
*D. gracilis*	DHMECN 2902	Ecuador	Manabí	El Aguacate	0.65348, -80.05190		43
*D. gracilis*	QCAZ 11427	Ecuador	Manabí	Jama Coaque	-0.11455, -80.12337		321
*D. gracilis*	QCAZ 4654	Ecuador	Manabí	Lalo Loor	-0.08337, -80.15004		75
*D. gracilis*	MHNG 1363.023	Ecuador	Manabí	Maicito	-0.27265, -79.57179		173
*D. gracilis*	MHNG 1363.024	Ecuador	Manabí	Maicito	-0.27265, -79.57179		173
*D. gracilis*	MHNG 1363.026	Ecuador	Manabí	Maicito	-0.27265, -79.57179		173
*D. gracilis*	MHNG 1363.027	Ecuador	Manabí	Maicito	-0.27265, -79.57179		173
*D. gracilis*	QCAZ 4649	Ecuador	Manabí	Reserva Jama Coaque	-0.11556, -80.12472		299
*D. gracilis*	MHNG 2453.019	Ecuador	Manabí	Zapallo Grande	0.78165, -78.98345		100
*D. gracilis*	QCAZ 14494	Ecuador	Pichincha	Cachaco–Lita	0.78886, -78.36794		1108
*D. gracilis*	MZUTI 1386	Ecuador	Pichincha	El Abrazo del Árbol	-0.00913, -78.81321		1064
*D. gracilis*	QCAZ 7532	Ecuador	Pichincha	El Monte	-0.06912, -78.76195		1316
*D. gracilis*	QCAZ 15718	Ecuador	Pichincha	Finca Ecológica Orongo	0.15304, -78.66737		1173
*D. gracilis*	MZUTI 3503	Ecuador	Pichincha	Mashpi lodge	0.16681, -78.88111		905
*D. gracilis*	QCAZ 15542	Ecuador	Pichincha	Rainforest Monterreal	0.01557, -78.88407		860
*D. gracilis*	QCAZ 7322	Ecuador	Pichincha	Road to Mindo	-0.03116, -78.75617		1638
*D. gracilis*	QCAZ 3693	Ecuador	Santo Domingo	8.5 km NW Santo Domingo	-0.17700, -79.21099		454
*D. gracilis*	QCAZ 3694	Ecuador	Santo Domingo	8.5 km NW Santo Domingo	-0.17700, -79.21099		454
*D. gracilis*	QCAZ 11238	Ecuador	Santo Domingo	Finca de Germán Cortez	-0.00027, -79.41194		194
*D. gracilis*	QCAZ 2040	Ecuador	Santo Domingo	La Perla	0.13417, -79.49432		132
*D. gracilis*	DHMECN 129	Ecuador	–	–	–	–	–
*D. gracilis*	MZUTI 4199	Ecuador	–	–	–	–	–
*D. indica*	MZUA.RE.0059	Ecuador	Morona Santiago	Rosa de Oro	–	–	–
*D. indica*	MHNG 2435.093	Ecuador	Orellana	Coca	-0.46167, -76.99310		253
*D. indica*	MHNG 2413.076	Ecuador	Orellana	Hacienda Primavera	-0.48689, -76.63581		267
*D. indica*	MZUTI 4735	Ecuador	Pastaza	Tzarentza	-1.35696, -78.05814		1355
*D. jamespetersi*	MZUA.RE.0147	Ecuador	Azuay	La Paz	-3.31481, -79.15166		3148
*D. jamespetersi*	MZUTI 5307	Ecuador	Azuay	Poetate	-3.41645, -79.26964		2269
*D. jamespetersi*	USNM 237040	Ecuador	Loja	0.5 km E of Loja	-3.99277, -79.18327		2263
*D. jamespetersi*	MHNG 2512.047	Ecuador	Loja	24 km S Loja	-4.22083, -79.24164		1562
*D. jamespetersi*	MHNG 2512.048	Ecuador	Loja	24 km S Loja	-4.22083, -79.24164		1562
*D. jamespetersi*	MHNG 2399.071	Ecuador	Loja	5 km E Loja	-3.98899, -79.16576		2610
*D. jamespetersi*	MHNG 2457.09	Ecuador	Loja	5 km E Loja	-3.98899, -79.16576		2610
*D. jamespetersi*	MHNG 2512.049	Ecuador	Loja	5 km E Loja	-3.98899, -79.16576		2610
*D. jamespetersi*	MHNG 2512.05	Ecuador	Loja	5 km E Loja	-3.98899, -79.16576		2610
*D. jamespetersi*	MHNG 2521.087	Ecuador	Loja	5 km E Loja	-3.98899, -79.16576		2610
*D. jamespetersi*	QCAZ 15100	Ecuador	Loja	Guachanamá	-4.04081, -79.88290		2787
*D. jamespetersi*	MHNG 2413.082	Ecuador	Loja	Loja	-4.00789, -79.21128		2166
*D. latifrontalis*	BMNH1946.1.20	Venezuela	Mérida	Aricagua	8.16162, -71.15776		1078
*D. klebbai*	QCAZ 1605	Ecuador	Napo	2 km E Borja	-0.41543, -77.83032		1608
*D. klebbai*	DHMECN 568	Ecuador	Napo	Borja	-0.42624, -77.84277		1698
*D. klebbai*	MHNG 2220.035	Ecuador	Napo	El Chaco	-0.33763, -77.80957		1595
*D. klebbai*	MHNG 2220.056	Ecuador	Napo	El Chaco	-0.33763, -77.80957		1595
*D. klebbai*	MHNG 2250.063	Ecuador	Napo	El Chaco	-0.33763, -77.80957		1595
*D. klebbai*	MHNG 2250.064	Ecuador	Napo	El Chaco	-0.33763, -77.80957		1595
*D. klebbai*	MZUTI 5412	Ecuador	Napo	Pacto Sumaco	-0.66377, -77.59895		1556
*D. klebbai*	MCZ 164674	Ecuador	Napo	Río Azuela	-0.14869, -77.65463		1402
*D. klebbai*	MCZ 164675	Ecuador	Napo	Río Azuela	-0.14869, -77.65463		1402
*D. klebbai*	USNM 286323	Ecuador	Napo	Río Azuela	-0.14869, -77.65463		1402
*D. klebbai*	MHNG 2220.038	Ecuador	Napo	San Rafael	-0.09669, -77.58995		1464
*D. klebbai*	MHNG 2220.039	Ecuador	Napo	San Rafael	-0.09669, -77.58995		1464
*D. klebbai*	MZUTI 63	Ecuador	Napo	Yanayacu	-0.60042, -77.89053		2110
*D. klebbai*	MHNG 2220.04	Ecuador	Sucumbíos	El Reventador	-0.04480, -77.52858		1476
*D. klebbai*	MHNG 2220.041	Ecuador	Sucumbíos	El Reventador	-0.04480, -77.52858		1476
*D. klebbai*	QCAZ 250	Ecuador	Sucumbíos	El Reventador	-0.04669, -77.52898		1428
*D. klebbai*	QCAZ 14281	Ecuador	Sucumbíos	La Bonita	0.47209, -77.54661		1953
*D. klebbai*	MHNG 2529.029	Ecuador	–	–	–	–	–
*D. klebbai*	ZSFQ D304	Ecuador	Napo	Cascada de San Rafael	-0.10007, -77.58034		1182
*D. georgejetti*	USNM 142595	Ecuador	Guayas	10 mi N of Guayaquil	-1.96418, -79.87988		5
*D. georgejetti*	QCAZ 9125	Ecuador	Guayas	Cerro Blanco	-2.17465, -80.02135		147
*D. georgejetti*	ENS 12817	Ecuador	Manabí	17 km NW Portoviejo	-1.00209, -80.31334		187
*D. georgejetti*	MZUTI 5411	Ecuador	Manabí	Cabuyal	-0.19698, -80.29059		15
*D. georgejetti*	QCAZ 10589	Ecuador	Manabí	El Aromo	-1.04665, -80.83276		295
*D. georgejetti*	DHMECN 11639	Ecuador	Manabí	Montecristi	-1.04694, -80.65766		136
*D. georgejetti*	MZUA.RE.0121	Ecuador	Manabí	El Aromo	-1.04665, -80.83276		295
*D. georgejetti*	MZUA.RE.0122	Ecuador	Manabí	El Aromo	-1.04665, -80.83276		295
*D. georgejetti*	DHMECN 11646	Ecuador	Manabí	Rocafuerte	-0.92371, -80.45212		19
*D. georgejetti*	ZSFQ D606	Ecuador	Manabí	Cerro La Mocora, foothill	-1.59817, -80.65431		308
*D. oligozonata*	MZUA.RE.0081	Ecuador	Azuay	Girón	-3.15891, -79.14755		2102
*D. oligozonata*	QCAZ 4472	Ecuador	Azuay	Granja Orgánica Susudel	-3.38885, -79.17847		2802
*D. oligozonata*	QCAZ 4492	Ecuador	Azuay	Susudel	-3.40543, -79.18378		2376
*D. oligozonata*	MZUA.RE.0240	Ecuador	Azuay	Via a Shaglli	-3.19178, -79.39623		2891
*D. oligozonata*	MZUA.RE.0020	Ecuador	–	–	–	–	–
*D. oligozonata*	MZUA.RE.0357	Ecuador	–	–	–	–	–
*D. oreas*	QCAZ 10140	Ecuador	Azuay	Luz María	-2.68548, -79.40992		1661
*D. oreas*	DHMECN 3478	Ecuador	Azuay	Naranjo Lanto	-2.92628, -79.39963		1847
*D. oreas*	DHMECN 7647	Ecuador	Azuay	Reserva Biológica Yunguilla	-3.22684, -79.27520		1748
*D. oreas*	DHMECN 7666	Ecuador	Azuay	Reserva Biológica Yunguilla	-3.22684, -79.27520		1748
*D. oreas*	MZUA.RE.0239	Ecuador	Azuay	San Rafael de Sharug	-3.27311, -79.54543		1593
*D. oreas*	MZUA.RE.0290	Ecuador	Azuay	San Rafael de Sharug	-3.27311, -79.54543		1593
*D. oreas*	QCAZ 9190	Ecuador	Azuay	Vía La Paz–Cuenca	-3.09021, -79.00800		2726
*D. oreas*	USNM 62797	Ecuador	Chimborazo	Pallatanga–Guayaquil	-2.07459, -78.98123		1404
*D. oreas*	USNM 62798	Ecuador	Chimborazo	Pallatanga–Guayaquil	-2.07459, -78.98123		1404
*D. oreas*	USNM 62800	Ecuador	Chimborazo	Pallatanga–Guayaquil	-2.07459, -78.98123		1404
*D. oreas*	DHMECN 10785	Ecuador	El Oro	Playa Limón	-3.50096, -79.74701		816
*D. oreas*	DHMECN 2572	Ecuador	El Oro	Reserva Buenaventura	-3.65467, -79.76794		524
*D. oreas*	MZUTI 3351	Ecuador	El Oro	Reserva Buenaventura	-3.64882, -79.75640		898
*D. oreas*	MZUTI 5415	Ecuador	El Oro	Reserva Buenaventura	-3.63432, -79.74985		1048
*D. oreas*	MZUTI 5418	Ecuador	El Oro	Reserva Buenaventura	-3.63370, -79.75040		1068
*D. oreas*	MHNG 2514.028	Ecuador	Loja	33 km E San Pedro	-3.97222, -79.25983		2493
*D. oreas*	MHNG 2521.084	Ecuador	Loja	6 km S Loja	-4.03770, -79.19975		2144
*D. oreas*	QCAZ 10068	Ecuador	Loja	Cazerío Balzones	-4.01502, -80.01635		1346
*D. oreas*	QCAZ 13875	Ecuador	Loja	Jimbura	-4.66668, -79.45322		2513
*D. oreas*	QCAZ 11290	Ecuador	Loja	Vía al Cerro Toledo	-4.38444, -79.15992		2214
*D. oreas*	QCAZ 678	Ecuador	Loja	Vilcabamba	-4.25792, -79.21962		1546
*D. oreas*	QCAZ 6020	Ecuador	Loja	Yangana–Vilcabamba	-4.32455, -79.20041		1742
*D. palmeri*	QCAZ 11411	Ecuador	Morona Santiago	9 de Octubre–Macas	-2.21820, -78.29920		1767
*D. palmeri*	QCAZ 5609	Ecuador	Morona Santiago	Chiguinda	-3.28125, -78.69829		2223
*D. palmeri*	DHMECN 11197	Ecuador	Morona Santiago	Concesión ECSA	-3.57524, -78.43609		1211
*D. palmeri*	QCAZ 13307	Ecuador	Morona Santiago	Laguna Chimerella	-2.07956, -78.20338		1795
*D. palmeri*	QCAZ 13304	Ecuador	Morona Santiago	Laguna Cormorán	-2.07153, -78.21590		1747
*D. palmeri*	QCAZ 13562	Ecuador	Pastaza	Tzarentza	-1.35696, -78.05814		1355
*D. palmeri*	QCAZ 4710	Ecuador	Tungurahua	3 km E Río Verde	-1.40249, -78.28369		1474
*D. palmeri*	AMNH 24126	Ecuador	Tungurahua	Abitagua	-1.41667, -78.16667		1353
*D. palmeri*	MZUTI 4804	Ecuador	Tungurahua	Agoyán	-1.39795, -78.38415		1661
*D. palmeri*	MZUA.RE.0044	Ecuador	Tungurahua	Baños	-1.39650, -78.42945		1847
*D. palmeri*	QCAZ 14071	Ecuador	Tungurahua	Baños	-1.39650, -78.42945		1847
*D. palmeri*	QCAZ 3288	Ecuador	Tungurahua	Baños	-1.39650, -78.42945		1847
*D. palmeri*	QCAZ 4710	Ecuador	Tungurahua	Caserío Machay	-1.40062, -78.28085		1531
*D. palmeri*	DHMECN 9229	Ecuador	Tungurahua	Chamanapamba	-1.40114, -78.39975		1808
*D. palmeri*	DHMECN 9230	Ecuador	Tungurahua	Chamanapamba	-1.40114, -78.39975		1808
*D. palmeri*	MZUTI 3956	Ecuador	Tungurahua	La Candelaria	-1.43051, -78.31246		1920
*D. palmeri*	AMNH 37939	Ecuador	Tungurahua	Palmera	-1.41613, -78.19663		1225
*D. palmeri*	DHMECN 9232	Ecuador	Tungurahua	Parque Juan Montalvo	-1.40005, -78.42070		1803
*D. palmeri*	QCAZ 13992	Ecuador	Tungurahua	Río Verde	-1.39406, -78.30405		1603
*D. palmeri*	QCAZ 4564	Ecuador	Tungurahua	Río Verde	-1.39406, -78.30405		1603
*D. palmeri*	DHMECN 12841	Ecuador	Tungurahua	Ulba	-1.39622, -78.39418		1702
*D. palmeri*	DHMECN 9219	Ecuador	Tungurahua	Vizcaya	-1.34789, -78.40518		2282
*D. palmeri*	QCAZ 6021	Ecuador	Zamora Chinchipe	18.2 km W Zamora	-3.97643, -79.02075		1609
*D. palmeri*	QCAZ 3001	Ecuador	Zamora Chinchipe	182 km Zamora–Loja	-3.95600, -79.02599		1665
*D. palmeri*	QCAZ 14338	Ecuador	Zamora Chinchipe	Estación San Francisco	-3.96128, -79.05556		1775
*D. palmeri*	QCAZ 12771	Ecuador	Zamora Chinchipe	Reserva Numbami	-4.17233, -78.95928		1615
*D. palmeri*	MZUTI 4971	Ecuador	Zamora Chinchipe	Reserva San Francisco	-3.97051, -79.07814		1850
*D. palmeri*	MZUTI 4975	Ecuador	Zamora Chinchipe	Reserva San Francisco	-3.97140, -79.07909		1730
*D. palmeri*	QCAZ 12772	Ecuador	Zamora Chinchipe	Reserva San Francisco	-3.97051, -79.07814		1850
*D. palmeri*	MZUTI 5419*	Ecuador	Zamora Chinchipe	Romerillos Alto	-4.23230, -78.94222		1547
*D. palmeri*	QCAZ 12510	Ecuador	Zamora Chinchipe	Zumba	-4.86517, -79.13384		1230
*D. palmeri*	MZUA.RE.0119	Ecuador	–	–	–	–	–
*D. palmeri*	BMNH 1946.1.2077	Peru	Cajamarca	Jaén	-5.72978, -78.84836		1438
*D. palmeri*	MCZ 17404	Peru	Cajamarca	Tabaconas	-5.31429, -79.29622		1892
*D. pavonina*	MZUA.RE.0198	Ecuador	Morona Santiago	Kushapuk	-3.04373, -78.03648		326
*D. pavonina*	QCAZ 5554	Ecuador	Morona Santiago	Tiink	-3.34389, -78.46805		730
*D. pavonina*	MHNG 2309.039	Ecuador	Napo	Archidona	-0.90856, -77.80814		571
*D. pavonina*	MHNG 2521.088	Ecuador	Napo	Tena	-0.98330, -77.81670		522
*D. pavonina*	MZUTI 4972	Ecuador	Zamora Chinchipe	Maycu	-4.38030, -78.74584		981
*D. peruana*	LSUMZ 27372	Peru	Amazonas	28 km SE Ingenio	-6.05753, -77.98919		2235
*D. peruana*	KU 212590	Peru	Amazonas	Pomacochas	-5.82155, -77.91692		2150
*D. peruana*	MCZ 178175	Peru	Cuzco	Amaibamba	-13.27703, -73.28636		1858
*D. peruana*	LSUMZ 27369–70	Peru	Cuzco	Bosque Aputinye	-12.92300, -72.67455		1502
*D. peruana*	KU 117109	Peru	Cuzco	Machu Picchu	-13.17104, -72.50585		2400
*D. peruana*	AMNH 147037	Peru	Cuzco	Paucartambo Mirador	-13.06972, -71.55527		1818
*D. peruana*	AMNH 147037	Peru	Cuzco	Paucartambo Mirador	-13.06972, -71.55527		1810
*D. peruana*	USNM 60718	Peru	Cuzco	Pucyura	-13.07450, -72.93437		2666
*D. peruana*	CORBIDI 11839	Peru	Cuzco	Rocotal	-13.10627, -71.57064		2004
*D. peruana*	SMF 20801	Peru	Cuzco	Santa Ana	-12.86755, -72.71670		1639
*D. peruana*	LSUMZ 45499	Peru	Huánuco	Playa Pampa	-9.95160, -75.69605		2091
*D. peruana*	BMNH 1946.1.2078	Peru	Pasco	Huancabamba	-10.42265, -75.51718		1775
*D. peruana*	USNM 299232	Peru	Puno	10 km NNE Ollachea	-13.78330, -70.46730		2598
*D. peruana*	USNM 299234	Peru	Puno	11 km NNE Ollachea	-13.78661, -70.47248		2601
*D. peruana*	USNM 299233	Peru	Puno	12 km NNE Ollachea	-13.78330, -70.46730		2598
*D. peruana*	AMNH 52444	Peru	San Martín	Cumbre Ushpayacu-Mishquiyacu	-6.99468, -76.03371		1279
*D. temporalis*	MZUTI 3331	Ecuador	Esmeraldas	Tundaloma Lodge	1.18317, -78.75245		74
*D. temporalis*	MHNG 2521.083	Ecuador	Imbabura	16 km W Lita	0.90235, -78.54504		799
*D. vagrans*	AMNH 63373	Peru	San Martín	Bellavista	-7.05346, -76.58928		316
*D. vermiculata*	MHNG 2521.085	Ecuador	Morona Santiago	69 km S Vilcabamba	-4.84920, -79.12731		1310
*D. vermiculata*	DHMECN 11197	Ecuador	Morona Santiago	Concesión ECSA	-3.57245, -78.46982		790
*D. vermiculata*	MHNG 2436.014	Ecuador	Napo	El Reventador	-0.04480, -77.52858		1476
*D. vermiculata*	MZUTI 5080	Ecuador	Pastaza	Kallana	-1.469629, -77.27838		325
*D. vermiculata*	QCAZ 13825	Ecuador	Pastaza	Sendero Higuerones	-4.11464, -78.96702		981
*D. vermiculata*	MZUTI 4738	Ecuador	Pastaza	Tzarentza	-1.35696, -78.05814		1355
*D. vermiculata*	MZUTI 3663	Ecuador	Zamora Chinchipe	Maycu	-4.20719, -78.63987		869
*D. vermiculata*	MZUA.RE.0261	Ecuador	Zamora Chinchipe	Nangaritza	-4.43169, -78.63869		1011
*D. oswaldobaezi*	QCAZ 14051	Ecuador	El Oro	Arenillas	-3.62110, -80.17513		41
*D. oswaldobaezi*	QCAZ 14060	Ecuador	El Oro	Guabillo	-3.60346, -80.18139		44
*D. oswaldobaezi*	MZUA.RE.0286	Ecuador	El Oro	Huaquillas	-3.54115, -80.08646		39
*D. oswaldobaezi*	QCAZ 10369	Ecuador	Loja	Quebrada El Faique	-4.17889, -80.04226		1004
*D. oswaldobaezi*	QCAZ 15108	Ecuador	Loja	Reserva La Ceiba-Pilares	-4.27502, -80.32805		534
*D. oswaldobaezi*	BMNH1935.11.3.108	Ecuador	Loja	Catamayo	-3.98064, -79.35928		1289
*D. oswaldobaezi*	MUSM 2192	Peru	Piura	Piura	-5.17882, -80.62231		32
*S. annulatus*	MZUTI 3034	Ecuador	Esmeraldas	Reserva Itapoa	0.51307, -79.13401		321
*S. bevridgelyi*	MZUA.RE.0424	Ecuador	Azuay	2 km N Palmales Nuevo	-3.65158, -80.09625		129
*S. bevridgelyi*	KU 152205	Ecuador	Azuay	30 KM E Pasaje	-3.31439, -79.57970		561
*S. bevridgelyi*	QCAZ 14446	Ecuador	Azuay	Ponce Enríquez–El Coca	-3.03197, -79.64615		1206
*S. bevridgelyi*	QCAZ 14444	Ecuador	Azuay	Proyecto Minas San Francisco	-3.30829, -79.47079		862
*S. bevridgelyi*	MZUA.RE.0142	Ecuador	Azuay	Sarayunga	-3.31431, -79.58069		552
*S. bevridgelyi*	MCZ R-17099	Ecuador	Chimborazo	Valle del Chanchán	-2.27383, -79.08735		697
*S. bevridgelyi*	DHMECN 11526	Ecuador	El Oro	Remolino	-3.56551, -79.91948		229
*S. bevridgelyi*	DHMECN 9483	Ecuador	El Oro	Reserva Buenaventura	-3.65467, -79.76794		524
*S. bevridgelyi*	MZUTI 3269	Ecuador	El Oro	Reserva Buenaventura	-3.65343, -79.76722		473
*S. bevridgelyi*	MZUTI 5416	Ecuador	El Oro	Reserva Buenaventura	-3.65467, -79.76794		524
*S. bevridgelyi*	AMNH 22092	Ecuador	Guayas	Reserva Ayampe	-1.65417, -80.81833		43
*S. bevridgelyi*	MCZ R-3564	Ecuador	Guayas	Río Daule	-1.87009, -80.00539		5
*S. bevridgelyi*	MZUA.RE.0328	Ecuador	Los Ríos	Jauneche	-1.33333, -79.58333		41
*S. bevridgelyi*	DHMECN 8976	Ecuador	Manabí	San Sebastián	-1.60002, -80.69974		602
*S. bevridgelyi*	DHMECN 10061	Ecuador	Manabí	Puerto López	-1.55598, -80.81200		3
*S. bevridgelyi*	ZSFQ D503	Ecuador	Manabí	Cerro La Mocora, tophill	-1.60379, -80.70191		818
*S. bevridgelyi*	CORBIDI 3791	Peru	Tumbes	El Caucho	-3.81438, -80.27101		379
*S. bevridgelyi*	CORBIDI 3792	Peru	Tumbes	El Caucho	-3.81438, -80.27101		379
*S. bevridgelyi*	CORBIDI 7894	Peru	Tumbes	El Caucho	-3.81844, -80.26856		478
*S. bevridgelyi*	CORBIDI 7994	Peru	Tumbes	El Caucho	-3.81244, -80.26716		481
*S. nebulatus*	MZUTI 4810	Ecuador	Cotopaxi	El Jardín de los Sueños	-0.83142, -79.21337		349
*S. nebulatus*	DHMECN 9585	Ecuador	Esmeraldas	Canandé	0.52580, -79.20880		310
*S. nebulatus*	DHMECN 5645	Ecuador	Esmeraldas	Lita–San Lorenzo	1.18236, -78.79528		42
*S. nebulatus*	MZUTI 3911	Ecuador	Esmeraldas	Reserva Itapoa	0.51307, -79.13401		321
*S. nebulatus*	DHMECN 5647	Ecuador	Esmeraldas	Tundaloma	1.18236, -78.75250		74
*S. nebulatus*	DHMECN 10312	Ecuador	Imbabura	Selva Alegre	0.26667, -78.58333		1299
*S. nebulatus*	USNM 285501	Ecuador	Los Ríos	Hacienda Cerro Chico	-0.62444, -79.42940		170
*S. nebulatus*	MZUA.RE.0174	Ecuador	Los Ríos	Macul	-1.12980, -79.65730		65
*S. nebulatus*	USNM 285498	Ecuador	Los Ríos	Río Palenque	-0.58333, -79.36667		173
*S. nebulatus*	USNM 285499	Ecuador	Los Ríos	Río Palenque	-0.58333, -79.36667		173
*S. nebulatus*	USNM 285500	Ecuador	Los Ríos	Río Palenque	-0.58333, -79.36667		173
*S. nebulatus*	DHMECN 2882	Ecuador	Manabí	Aguacate	0.65348, -80.05190		43
*S. nebulatus*	MZUTI 5342	Ecuador	Manabí	Jama Coaque	-0.11556, -80.12472		299
*S. nebulatus*	DHMECN 1704	Ecuador	Pichincha	Curipogio	0.13112, -78.67632		1171
*S. nebulatus*	USNM 283534	Ecuador	Santo Domingo	Rancho Santa Teresita	-0.25277, -79.37946		288

### Systematics of the *Dipsas peruana* complex.

Based on differences in coloration and the topology of the molecular phylogeny obtained here (Fig. 3), we partition *Dipsas peruana* sensu Harvey and Embert (2008) into four allopatric species. This includes restriction of *D. peruana* to Peruvian-Bolivian populations, the resurrection of *D. palmeri* for populations ranging from northern Peru to central Ecuador, the description of a new species for northern Ecuador, and the resurrection of *D. latifrontalis* for populations in Colombia and Venezuela (Fig. 4).

#### Dipsas klebbai sp. n.

Taxon classificationAnimaliaSquamataDipsadidae

http://zoobank.org/83EDA906-74F7-4D2F-8E6A-59B23964897C

Figs 1l, m , 15 , 16

Dipsas peruana Harvey & Embert, 2008: 79 (part).

##### Proposed standard English name.

Klebba’s Snail-Eater

##### Proposed standard Spanish name.

Caracolera de Klebba

##### Holotype.

MZUTI 5412 (Figs 15, 16), adult male collected by Phillip Torres on April 28, 2016 at Pacto Sumaco, province of Napo, Ecuador (S0.66377, W77.59895; 1556 m).

##### Paratypes.

DHMECN 568, adult female collected by Thomas Begher on 1980 at Borja, province of Napo, Ecuador (S0.42054, W77.84104; 1717 m). MCZ 164674–75, two adults of undetermined sex collected by Giovani Onore on June 01, 1983 at Río Azuela, province of Napo, Ecuador (S0.148693, W77.65463; 1402 m). MHNG 2220.035, 2220.056, 2250.063, 2250.064, one juvenile female and three adult males, respectively, collected by Giovani Onore on 1984 at El Chaco, province of Napo, Ecuador (S0.33763, W77.80957; 1595 m). MHNG 2220.038–039, adult female and adult male, respectively, collected by Giovani Onore on November 1984 at San Rafael, province of Napo, Ecuador (S0.09669, W77.58995; 1464 m). MHNG 2220.04, 2220.041, adult females collected by Giovani Onore on May 1984 at El Reventador, province of Napo, Ecuador (S0.04480, W77.52858; 1476 m). MZUTI 63, adult male collected by Alejandro Arteaga on August 08, 2011 at Yanayacu, province of Napo, Ecuador (S0.60042, W77.89053; 2110 m). MNHG 2529.029, adult female collected by Eugen Kramer on February 22, 1992 at Napo province, Ecuador. QCAZ 12488, collected by Pablo Medrano on March 02, 2015 at Río Quijos, province of Napo, Ecuador (S0.45224, W77.94249; 1929 m). QCAZ 12600, collected by Pablo Medrano on March 27, 2014 at Santa Rosa, province of Napo, Ecuador (S0.39630, W77.82343; 1113 m). QCAZ 13124, collected by Fabián Vallejo on November 21, 2014 at Las Palmas, province of Napo, Ecuador (S0.54691, W77.87762; 1903 m). QCAZ 14281, adult male collected by Andrea Narváez on December 02, 2016 at La Bonita, province of Sucumbíos, Ecuador (N0.47209, W77.54661; 1953 m). QCAZ 1496, collected on October 18, 1992 at Sardinas, province of Napo, Ecuador (S0.38484, W77.83782; 1641 m). QCAZ 1605, adult male collected by Victor Utreras on February 04, 1992 at 2 km E Borja, province of Napo, Ecuador (S0.41543, W77.83032; 1608 m). QCAZ 250, adult male collected at El Reventador, province of Napo, Ecuador (S0.04480, W77.52858; 1476 m). QCAZ 358–59, collected on January 10, 1984 at Cascada de San Rafael, province of Napo, Ecuador (S0.10354, W77.58337; 1246 m). QCAZ 4500, collected by Estefanía Boada on August 01, 2011 at Hostería Cumandá, province of Napo, Ecuador (S0.45249, W77.88071; 1856 m). QCAZ 9696, collected by Steven Poe on August 04, 2009 at 2.3 km N of turnoff to Baeza, province of Napo, Ecuador (N0.45236, W77.88212; 1840 m). USNM 386323, adult female collected on February 24, 1979 at Río Azuela, province of Napo, Ecuador (S0.148693, W77.65463; 1402 m). ZSFQ D304, female collected by Jean-Marc Touzet and Diego F. Cisneros-Heredia at Cascada de San Rafael, province of Napo, Ecuador (S0.10007, W77.58034; 1182 m).

##### Diagnosis.

*Dipsas klebbai* is placed in the genus *Dipsas* based on phylogenetic evidence (Fig. 3), and the absence of a labial that is noticeably higher than other labials and in contact with the postocular, primary and secondary temporals. The species differs from all described species of *Dipsas* based on the following combination of characters: (1) 15/15/15 smooth dorsals with enlarged vertebral row (1.5–1.8 times as wide as adjacent rows); (2) one loreal and one preocular in contact with orbit; (3) 9–11 supralabials with (usually) 4^th^ to 6^th^ contacting orbit; (4) one pair of infralabials in contact behind symphysial; (5) 181–201 ventrals in males, 187–194 in females; (6) 99–123 divided subcaudals in males, 98–106 in females; (7) dorsal and ventral ground color light brown with various degrees of fine black speckling and 27–36 dark brown to black, cream-edged oblong blotches that are longer that interspaces and become smaller towards the tail (Fig. 2m, n); on first half of body, the dark bands meet ventrally to form full body rings; on second half they fail to meet ventrally; head black with different degrees of whitish edging on the labial scales, and a thin (1–2 scales long) cream to light brown irregular nuchal collar; dorsal blotches usually incomplete ventrally, extending far onto ventrals and occasionally fusing midventrally; cream edges of neighboring blotches fused in first 6–9 blotches; (8) 401–749 mm SVL in males, 525–630 mm in females; (9) 169–330 mm TL in males, 209–240 mm in females.

**Figure 9.**
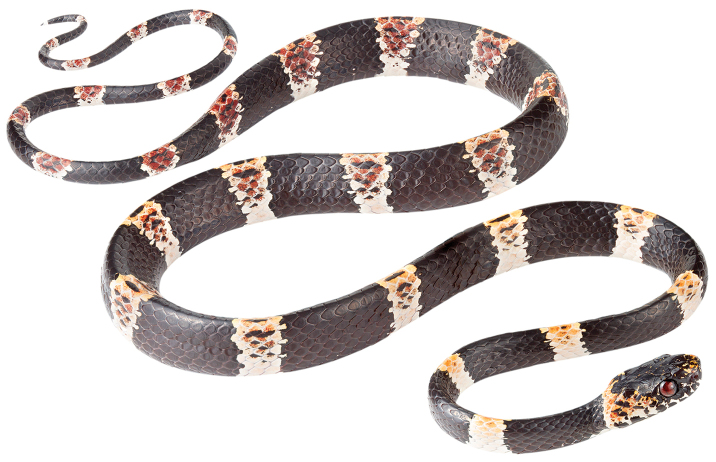
Adult male holotype of *Dipsas bobridgelyi*. MZUTI 5417.

**Figure 10.**
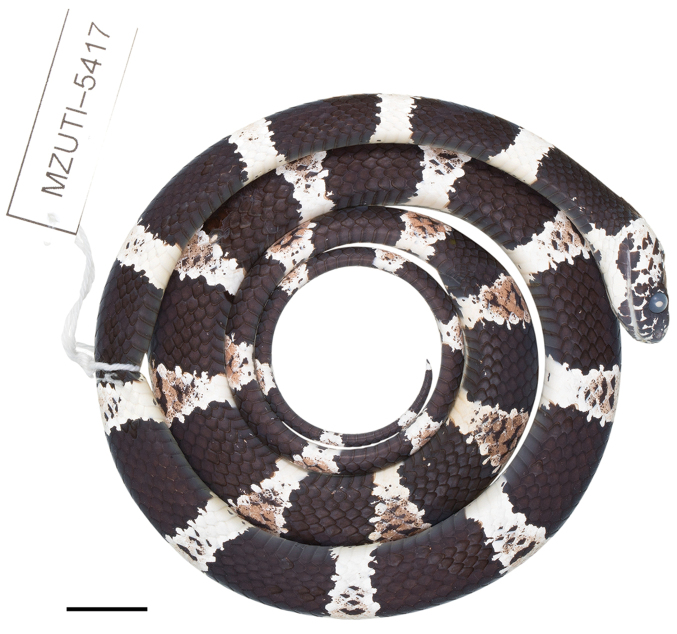
Adult male holotype of *Dipsas bobridgelyi*. MZUTI 5417. Scale bar: 1 cm.

##### Comparisons.

*Dipsas klebbai* is compared to species previously subsumed under *D. peruana*: *D. latifrontalis*, *D. palmeri*, and *D. peruana*. From *D. latifrontalis* (Fig. 1n) and *D. palmeri* (Figs 1r, s), it differs in having longer oblong to rectangular body blotches up to 7–13 vertebral scales long (vs. fewer than 8 vertebral scales long in *D. latifrontalis* and *D. palmeri*) that are also longer than the interspaces (Fig. 1l, m). Specimens of *D. klebbai* can be separated from specimens of *D. peruana*, with the exception of BMNH 1946.1.2078, based on the presence of the following characteristics (condition of *D. peruana* in parentheses): posterior body blotches twice to four times as long as interspaces (vs. posterior body blotches ca. equal in length or marginally longer than interspaces); interspaces never completely obscured by black pigment (vs. completely melanized in some specimens); dorsal surface of head black (vs. dark brown with dingy cream reticulations); dorsal body blotches fused ventrally on the first half of the body (vs. rarely fused); longest body blotch at least 7 vertebral scales long (vs. longest body blotch 4–7 vertebral scales long). Genetic divergence in a 684 bp long fragment of the mitochondrial Cytb gene between *D. klebbai* and *D. palmeri* is 8.2–9.2%, whereas intraspecific distances are less than 1.1% in both species. For the same fragment, the distance between *D. klebbai* and *D. peruana* is 10.7–11.0%.

##### Description of holotype.

Adult male, SVL 608 mm, tail length 262 mm (43% SVL); head length 20.3 mm (3% SVL) from tip of snout to commissure of mouth; head width 12.7 mm (62% head length) taken at broadest point; snout-orbit distance 5.4 mm; head distinct from neck; snout short, blunt in dorsal and lateral outline; rostral 4.0 mm wide, broader than high; internasals 2.6 mm wide, as broad as long; prefrontals 3.9 mm wide, broader than long, excluded from entering orbit by preocular; supraocular 4.3 mm long, broader than long; frontal 4.5 mm long, hexagonal, in contact with prefrontals, supraoculars, and parietals; parietals 6.6 mm long, longer than broad; nasal divided, in contact with first two supralabials, loreal, prefrontal, internasal, and rostral; loreal 2.6 mm long, slightly longer than high, entering orbit; eye diameter 4.5 mm; pupil semi-elliptical; one preocular; two postoculars; temporals 2+2; ten supralabials, 5^th^ and 6^th^ contacting orbit; symphysial separated from chinshields by the first pair of infralabials; 14 infralabials, 2–7 contacting chinshields; anterior pair of chinshields longer than broad, posterior pair broader than long; dorsal scales in 15/15/15 rows, smooth, without apical pits; 188 ventrals; 116 divided subcaudals; cloacal plate single.

##### Natural history.

At night (21h53–02h13), specimens of *Dipsas klebbai* have been found active during or after light rain on arboreal vegetation 50–500 cm above the ground in a variety of environments ranging from primary montane cloud forests and evergreen montane forests to silvopastures and forest borders, occasionally close to rivers. By day, individuals have been found hidden underground in pastures or among shrubs in rural gardens, or coiled on leaves at 300 cm above the ground. At dusk, after warm days, individuals of *Dipsas klebbai* have been seen crossing roads. QCAZ 13124 laid six eggs on December 2014. Five eggs were found inside a rotten trunk at El Chaco, province of Napo Ecuador.

##### Distribution.

Endemic to the eastern slopes of the Ecuadorian Andes in the provinces of Napo and Sucumbíos at elevations between 1246 and 2120 m (Fig. 4).

##### Etymology.

Named after Casey Klebba, in recognition of his appreciation of and passion for Andean wildlife, and his invaluable support of AA’s field expeditions to remote areas of Ecuador. After a visit to Peru in 2011, Casey became an active supporter of conservation and scientific projects in Ecuador.

##### Conservation status.

All known localities of occurrence for *Dipsas klebbai* fall within the limits or within the buffer zone of the following protected areas: Parque Nacional Cayambe Coca, Parque Nacional Sumaco Napo Galeras, Reserva Ecológica Antisana, and Reserva Ecológica Cofán Bermejo. Furthermore, the species is common in degraded environments, which suggests a degree of tolerance for habitat modification. For these reasons, and because it does not meet the criteria (IUCN 2001) for qualifying in a threatened category, we here list it as Least Concern following IUCN guidelines.

##### Remarks.

In their revision of *Dipsas peruana*, Harvey and Embert (2008) included specimens of *D. klebbai*. However, they found no characters that could diagnose these specimens from the rest of Ecuadorian and Peruvian specimens of *D.* “*peruana*” in order to establish species boundaries. They also grouped the then valid *D. boettgeri*, *D. latifrontalis*, and *D. polylepis* under *D. peruana*. The authors were right to point out that the different populations cannot be separated based on characters of lepidosis. However, they did not include molecular data in their analyses, and also failed to notice the geographically structured differences in the length of the body blotches and their relationship to the length of the interspaces.

**Figure 11.**
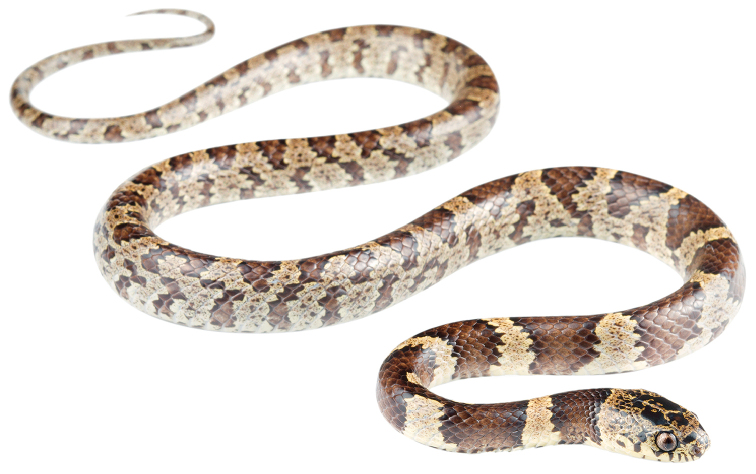
Adult male holotype of *Dipsas georgejetti*. MZUTI 5411.

**Figure 12.**
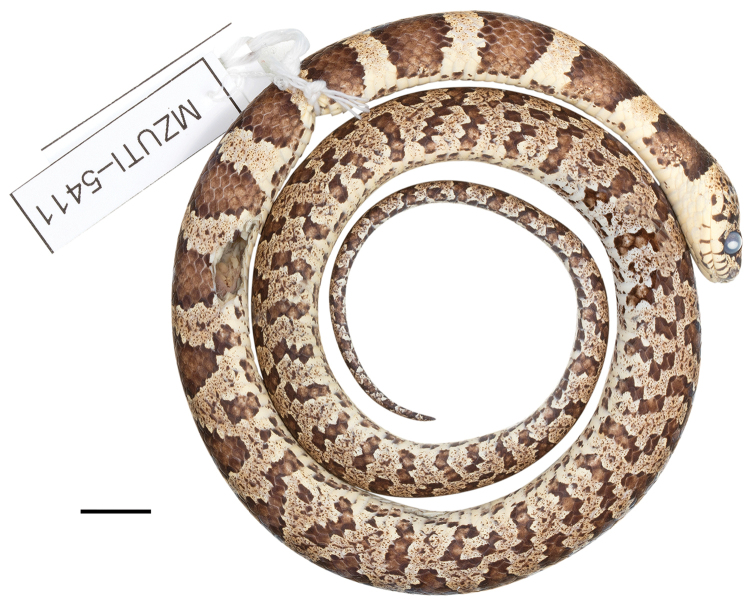
Adult male holotype of *Dipsas georgejetti*. MZUTI 5411. Scale bar: 1 cm.

#### Dipsas palmeri

Taxon classificationAnimaliaSquamataDipsadidae

(Boulenger, 1912)

Fig. 1r, s

Leptognathus palmeri Boulenger, 1912: 422. Holotype BMNH, a male from El Topo, province of Tungurahua, Ecuador.
Leptognathus latifasciatus Boulenger, 1913: 72. Holotype BMNH 1946.1.2007, a juvenile male from Upper Marañón, department of Cajamarca, Peru.
Dipsas peruana Harvey & Embert, 2008: 79 (part).

##### Proposed standard English name.

Palmer’s Snail-Eater

##### Proposed standard Spanish name.

Caracolera de Palmer

##### Diagnosis.

*Dipsas palmeri* differs from all described species of *Dipsas* based on the following combination of characters: (1) 15/15/15 smooth dorsals with enlarged vertebral row; (2) one loreal and one preocular in contact with orbit; (3) 8–10 supralabials with (usually) 4^th^ to 6^th^ contacting orbit; (4) one pair of infralabials in contact behind symphysial; (5) 172–202 ventrals in males, 181–200 in females; (6) 91–118 divided subcaudals in males, 86–102 in females; (7) dorsal and ventral ground color light brown with various degrees of fine black speckling and with 32–41 brown to blackish, white-edged circular blotches that are longer than interspaces in the first half of the body, but shorter in the second half (Figs 1r, s); adult head gray with different degrees of whitish edging on the labial scales, and a thin (1–2 scales long) white to light grayish brown irregular parietal collar; dorsal blotches incomplete ventrally, extending marginally onto ventrals but not fusing midventrally; (8) 215–907 mm SVL in males, 642–1187 mm in females; (9) 78–390 mm TL in males, 246–298 mm in females.

##### Comparisons.

*Dipsas palmeri* is compared to species previously subsumed under *D. peruana*: *D. latifrontalis*, *D. klebbai* (Fig. 1l, m), and *D. peruana*. From *D. latifrontalis* (Fig. 1n), it differs in having the first 19–35 dorsal blotches edged with white or cream, vs. the first 9–10 in *D. latifrontalis*. The only known adult of *D. latifrontalis* photographed in life has bronze interspaces (Fig. 1n), a coloration not seen in any adult of *D. palmeri*. From *D. klebbai*, it differs in having shorter blotches (longest blotch up to 3–7 vertebral scales long) that are circular (instead of oblong) and that are only longer than the interspaces on the first half of the body. From *D. peruana*, it differs in having dorsal blotches that are shorter than interspaces on posterior half of the body, and in lacking melanized interspaces in adult individuals.

##### Distribution.

Eastern slopes of the Ecuadorian and Peruvian Andes south of the Jatunyacu–Napo river valley in Ecuador and north of the Huancabamba depression at elevations between 1211 and 2282 m (Fig. 4).

##### Conservation status.

An estimated 31 out of the 42 known localities of occurrence for *Dipsas palmeri* are located within the limits or the buffer area of the following protected areas: Bosque Protector del Alto Nangaritza, Parque Nacional Llanganates, Parque Nacional Podocarpus and Parque Nacional Sangay. Furthermore, the presence of the species in degraded environments suggests a degree of tolerance for habitat modification. For these reasons, and because it does not meet the criteria for qualifying in a threatened category, we here list it as Least Concern following IUCN guidelines.

##### Remarks.

Neither Peters (1960) nor Harvey and Embert (2008) recognized the geographic morphological distinctiveness of *Dipsas palmeri* from Ecuador and Peru. Certainly, *D. palmeri* is most similar in coloration and lepidosis to *D. latifrontalis* (Fig. 1n) from Venezuela, and that is why Peters considered them synonyms. However neither Peters (1960) nor Harvey and Embert (2008) saw live specimens of *D. latifrontalis* in order to recognize the differences in life color pattern between the two species.

Two other junior synonyms of *Dipsas peruana* are *D. latifasciata* and *D. polylepis*, both of which occur in Peru (Fig. 4). Of these, only the latter must remain a synonym of *D. peruana*; the former should be transferred to the synonymy of *D. palmeri*, as defined here. Examination of photographs of the specimen of *D. latifasciata* (BMNH 1946.1.2077) reveals this species has dorsal blotches shorter than interspaces on posterior half of the body, a character seen in *D. palmeri* but not in *D. peruana*. The holotype was collected by A. E. Pratt in “Upper Marañón”, with no further specific locality mentioned. However, the type locality can be restricted to the immediate environs of the town of Jaén, as the “Upper Marañón” is considered the segment of the Marañón river that goes from the town of Jaén until the river meets the Santiago River. Additionally, in a letter to his wife in 1913, the explorer explains how he crossed the Ecuadorian Andes and arrived at the town of Jaén in northern Peru, where he stayed and collected specimens for the BMNH before proceeding to Iquitos along the Marañón river, with no mention of visiting any locality east of the river at elevations where *D. palmeri* and *D. peruana* are known to occur. Harvey and Embert (2008) pointed out that the Huancabamba depression could be a geographic barrier separating species within the *D. peruana* complex, but they did not find evidence to support this view. Our results suggest that the Huancabamba depression is a major geographic barrier separating *D. palmeri* (north) from *D. peruana* (south).

#### Dipsas peruana

Taxon classificationAnimaliaSquamataDipsadidae

(Boettger, 1898)

Leptognathus peruana Boettger, 1898: 128. Holotype SMF 20801, a female from Santa Ana, department of Cuzco, Peru.
Leptognathus boettgeri Werner, 1901: 11. Holotype MTKD D 1671 M, a female from Chanchamayo, department of Junín, Peru.
Leptognathus boliviana Werner, 1909: 240. Holotype ZMH, a female from department of Beni, Bolivia.
Leptognathus polylepis Boulenger, 1912: 422. Holotype BMNH 1946.1.2078, a female from Huancabamba, department of Pasco, Peru.

##### Proposed standard English name.

Peruvian Snail-Eater

##### Proposed standard Spanish name.

Caracolera Peruana

##### Diagnosis.

*Dipsas peruana* differs from all described species of *Dipsas* based on the following combination of characters: (1) 15/15/15 smooth dorsals with moderately enlarged vertebral row; (2) one loreal and one preocular in contact with orbit; (3) 8–9 supralabials with 4–6 or 3–5 contacting orbit; (4) one pair of infralabials in contact behind symphysial; (5) 177–200 ventrals in males, 180–203 in females; (6) 75–127 divided subcaudals in males, 79–105 in females; (7) dorsal and ventral ground color brown to dark brown (light brown in juveniles) with 33–43 blackish brown to complete black, white to cream edged circular to vertically elliptical blotches that are longer than interspaces; head dark brown with dingy cream reticulations and different degrees of whitish edging on the labial scales, and a thin (1–3 scales long) white to light grayish brown irregular nuchal collar; dorsal blotches extending marginally onto ventrals and rarely fusing midventrally; (8) 199 mm SVL in males, 610–725 mm in females; (9) 85 mm TL in males, 155–241 mm in females.

##### Comparisons.

*Dipsas peruana* *sensu stricto* is compared to species previously subsumed under *D. peruana* *sensu lato*: *D. latifrontalis*, *D. palmeri*, and *D. klebbai*. From *D. latifrontalis* and *D. palmeri*, it differs in having dorsal blotches along the entire body similar in length or longer than interspaces (shorter than interspaces in *D. latifrontalis* and *D. palmeri*), and in having melanized interspaces in some adult individuals. With the exception of BMNH 1946.1.2078, specimens of *D. peruana* can be separated from specimens of *D. klebbai* by possessing at least one of the following characteristics: posterior body blotches similar in length or marginally longer than interspaces (twice to four times as long in *D. klebbai*); short circular to vertically elliptical body blotches usually only up to 4–7 vertebral scales long; melanized interspaces; dorsal surface of the head not completely black; and dorsal body blotches rarely fused ventrally.

##### Distribution.

Eastern slopes of the Peruvian and Bolivian Andes south of the Huancabamba depression at elevations between 1279 and 2671 m (Fig. 4).

**Figure 13.**
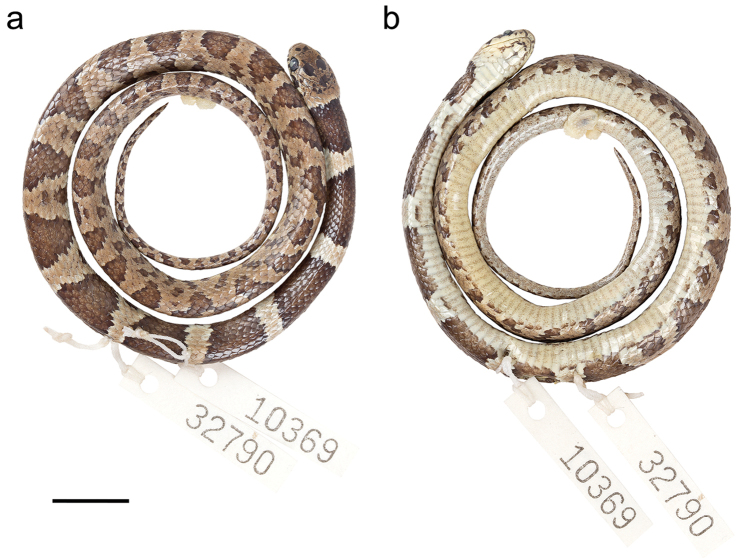
Adult female holotype of *Dipsas oswaldobaezi* QCAZ 10369 in **a** dorsal and **b** ventral view. Scale bar: 1 cm.

**Figure 14.**
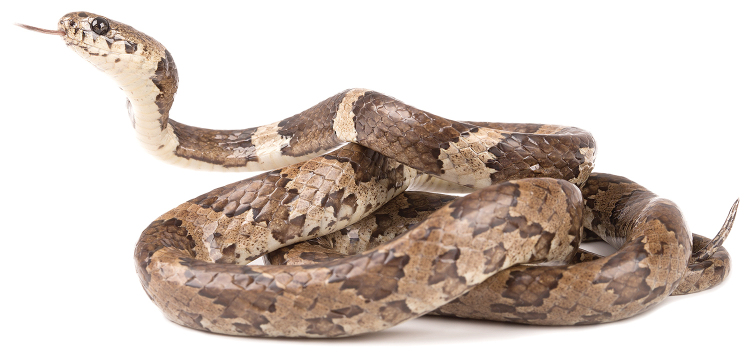
Adult female paratype of *Dipsas oswaldobaezi*. QCAZ 15108.

#### Dipsas latifrontalis

Taxon classificationAnimaliaSquamataDipsadidae

(Boulenger, 1905)

Leptognathus latifrontalis Boulenger, 1905: 561. Holotype BMNH 1946.1.20.98, a female from Aricagua, state of Mérida, Venezuela.
Dipsas peruana Harvey & Embert, 2008: 79 (part).

##### Proposed standard English name.

Broad-fronted Snail-Eater

##### Proposed standard Spanish name.

Caracolera frentona

##### Diagnosis.

*Dipsas latifrontalis* differs from all described species of *Dipsas* based on the following combination of characters: (1) 15/15/15 smooth dorsals with moderately enlarged vertebral row; (2) one loreal and one preocular in contact with orbit; (3) 8–10 supralabials with 3^rd^ to 6^th^ contacting orbit; (4) one pair of infralabials in contact behind symphysial; (5) 192 ventrals in one male (CVULA 7883), 194 in the female holotype; (6) 109 divided subcaudals in the single male, 95 in the female holotype; (7) dorsal and ventral ground color bronze (light brown in juveniles) with 32–36 dark reddish brown to black, circular to vertically elliptical blotches that are longer than interspaces and white to cream edged on first half of body; head grayish brown to black with different degrees of whitish edging on the labial scales, and with or without a thin (1–2 scales long) dingy white irregular nuchal collar; dorsal blotches extending marginally onto ventrals and occasionally fusing on the anterior part of the body; (8) 800 mm SVL in the holotype female; (9) 220 mm TL in the holotype female.

##### Comparisons.

*Dipsas latifrontalis* is compared to species previously subsumed under *D. peruana*: *D. palmeri*, *D. peruana*, and the herein described *D. klebbai*. From *D. palmeri*, it differs in having the first 9–10 dorsal blotches edged with white or cream, vs. the first 19–35 in *D. palmeri*. The only known adult of *D. latifrontalis* photographed in life has bronze interspaces (Fig. 1n), a coloration not seen in any adult of *D. palmeri* (see also Remarks below). From *D. klebbai*, it differs in having shorter blotches (longest blotch up to 6–8 vertebral scales long) that are circular (instead of oblong) and that are only longer than the interspaces on the first half of the body. From *D. peruana*, it differs in having dorsal blotches in posterior half of the body shorter than interspaces, and in lacking melanized interspaces in adult individuals.

##### Distribution.

Known only from two localities in the Venezuelan Andes and one in the Northern Colombian Andes at elevations between 1000 and 1400 m (Fig. 4).

##### Remarks.

Neither Peters (1960) nor Harvey and Embert (2008) examined the holotype of *Dipsas latifrontalis*, and they used Boulenger (1905) description to assign specimens of *D. palmeri* and *D. peruana*, respectively, to *D. latifrontalis*. We examined pictures of the holotype of *D. latifrontalis* from the BMNH, provided to us by César L. Barrio-Amorós. In coloration, the holotype is nearly identical to the uncollected adult presented in Figure 1n (San Isidro, Barinas province, Venezuela), with faint cream edging restricted to blotches 1–9, and indistinct blotches on the posterior part of the body. The previously only known photograph of a *D. latifrontalis* is of a juvenile from the same location as the specimen in Figure 1n (Rivas et al. 2012).

All *Dipsas latifrontalis* depicted in Lotzkat et al. (2008) and Natera-Mumaw et al. (2015) refer to a different species related to the *D. incerta* group, except for the holotype of *D. latifrontalis* BMNH 1946.1.20.98 (formerly 1905.5.31.76).

**Figure 15.**
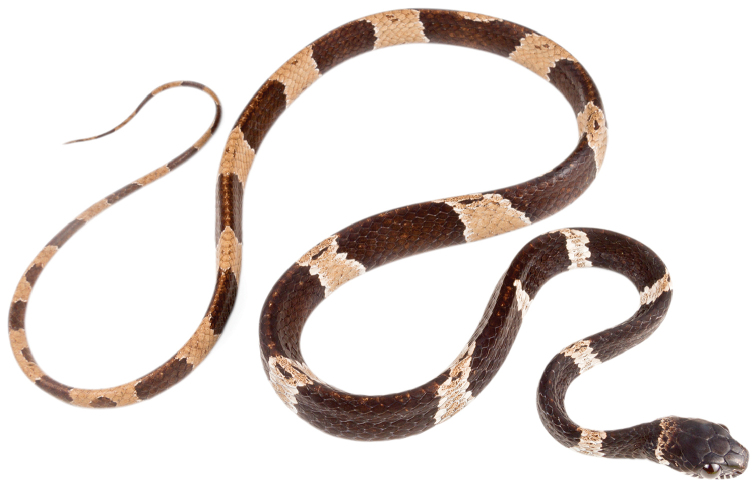
Adult male holotype of *Dipsas klebbai*. MZUTI 5412.

**Figure 16.**
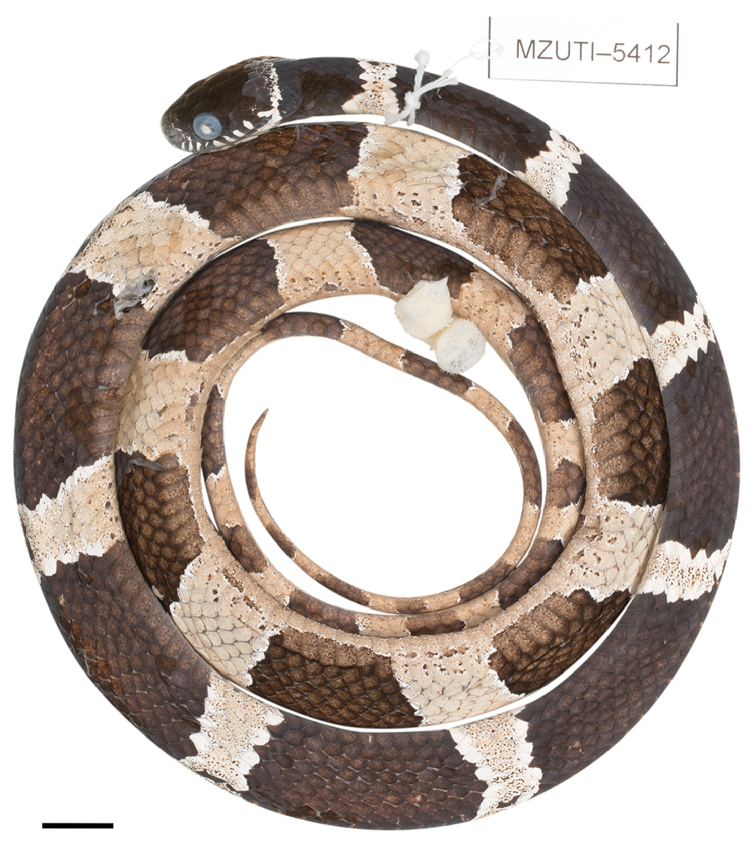
Adult male holotype of *Dipsas klebbai*. MZUTI 5412. Scale bar: 1 cm.

## Discussion

Higher-level relationships within Dipsadini are still far from being resolved. The monotypic *Plesiodipsas perijanensis* was not included in our analysis or other recent molecular phylogenies. The species of *Dipsas*+*Sibynomorphus* and *Sibon* included here form monophyletic groups, but this is not the case for the genus *Tropidodipsas*, for which *T. sartorii* and *T. fasciata* + *T. fischeri* are the successive sister lineages of *Dipsas*+*Sibynomorphus* and *Sibon* (Fig. 3). This arrangement mirrors the results of Sheehy’s (2012) unpublished PhD thesis, which presented evidence that groups consisting of *T. sartorii*, *T. annulifera*, *T. fischeri*, *T. philippii*, and *T. fasciatus*, as well as several new species of *Tropidodipsas* were not each other’s closest relatives, and some merited recognition as distinct genera. Sheehy (2012) also presented phylogenetic evidence that *Sibon sanniolus* and *Dipsas gaigeae* do not belong to their nominal genera. Instead, each is more closely related to *Tropidodipsas* *sensu stricto* (*D. gaigeae*) or “*T.*” *sartorii* + *Geophis* + “*T.*” *annulifera* (*S. sanniolus*) than any species of *Dipsas* or *Sibon*.

Decades ago, Parker (1926) and Smith and Taylor (1945) suggested that *Sibynomorphus* and *Dipsas* were synonyms. More recently, Zaher et al. (2009), Grazziotin et al. (2012), and Sheehy (2012) recognized that *Dipsas* is paraphyletic with respect to *Sibynomorphus*, a conclusion we corroborate based on the results of our ML molecular phylogeny. In fact, members of former *Sibynomorphus* fall into four different clades across the phylogeny of *Dipsas*. In general, we suggest that the former *Sibynomorphus* species represent cases of convergent evolution; apparently from within several independent *Dipsas* clades or they represent an ancient morphotype successfully persisting through today.

Additionally, many traditional infrageneric groups are either non-monophyletic, or poorly supported and weakly placed. We recognize that this may reflect inadequate sampling of taxa (only 43 of 77 species are included) or characters (only four mtDNA and one nuclear locus were used). From the eight *Dipsas* species groups recognized by Harvey (2008) (Table 1), we only found phylogenetic support for the *D. articulata* and *D. indica* species groups. Two groups of species that are monophyletic in our molecular phylogeny and are similar in coloration and lepidosis are: 1) *D. georgejetti* + *D. oligozonata* + *D. oswaldobaezi* + *D. williamsi*, and 2) *D. klebbai* + *D. palmeri* + *D. peruana*. The sampled members of the *D. oreas* group are monophyletic if *D. andiana* is placed in this group, as it is the strongly supported (in both BI and ML analyses) sister taxon of *D. oreas*. We therefore place *D. andiana* in the *D. oreas* group and propose that the same be done for the morphologically similar *D. nicholsi* from Panama.

*Dipsas bobridgelyi* is most similar in coloration to *D. gracilis* (Fig. 1h, i). These species are recovered as sister taxa in our phylogenetic analyses (Fig. 3) and have non-overlapping, but adjacent distribution ranges in western Ecuador (Fig. 4). This scenario suggests a parapatric speciation event, as the distribution of *D. gracilis* is congruent with Chocoan evergreen forest in northwestern Ecuador whereas the distribution of *D. bobridgelyi* is congruent with Tumbesian semi-deciduous forests in southwestern Ecuador.

Although we did not examine MUSM 17589 from Tumbes department, Peru, the description of the coloration and head scales of this specimen provided by Cadle (2005) and Harvey (2008) suggests that it is a *Dipsas bobridgelyi*, rather than a *D. gracilis*, as was originally suggested by both authors before the description of *D. bobridgelyi* herein. There is no other voucher of *D. gracilis* from Peru and it is unlikely that two morphologically and phylogenetically, and likely also ecologically very close species, occur in sympatry. Hence, from a biogeographic perspective, we suggest *D. gracilis* does not occur in Peru and that all specimens from south of the southern limit of *D. gracilis* in southwestern Ecuador and adjacent northwestern Peru represent *D. bobridgelyi*.

Peters (1960) recognized a geographic morphological structure within the widely distributed Sibon nebulatus when he defined the subspecies nebulatus, *leucomelas*, *hartwegi*, and *popayanensis*. Here, our genetic results corroborate that *S. nebulatus leucomelas* from Ecuador and *S. nebulatus hartwegi* are distinct from the two Central American samples from Belize and northeastern Costa Rica, a divergence already put forward by Sheehy (2012). Yet, *S. nebulatus* is paraphyletic with respect to both *S. dunni* and *S. bevridgelyi*, which group with *S. nebulatus leucomelas* from Ecuador. Elevation of the two subspecies *S. nebulatus leucomelas* and *S. nebulatus hartwegi* to full species status would resolve this paraphyly. However, we refrain from taking this step because our sample size for *S. nebulatus hartwegi* is small, even though plenty of photographic data from references (e.g., Natera-Mumaw et al. 2015) and online sources confirm that long nuchal bands and often brownish color pattern are typical of *S. nebulatus hartwegi* occurring from Medellin, Colombia, east into Venezuela. In addition, the supposedly diagnostic darker ground color of *S. nebulatus leucomelas* with copious blackish stippling of the interspaces and head (Peters 1960) is not exclusive of this subspecies. There is ample evidence (photographic vouchers, preserved specimens, online photo sources) that this color pattern is rather consistent in *S. n. nebulatus* from Nicaragua through Panama, and can even be observed in single specimens as far as the northern limit of the species in Mexico. Furthermore, we have no genetic data of *S. nebulatus* from southern Costa Rica, Panama, and Colombia, which could confirm a clear split between two species, rather than a gradient of two intergrading subspecies.

*Sibon bevridgelyi* and *S. nebulatus leucomelas* were not recovered as sister taxa in our phylogenetic analyses (Fig. 3), despite being similar in coloration and lepidosis, and having adjacent marginally overlapping distribution ranges in western Ecuador (Fig. 8), a pattern that would suggest an allopatric speciation event. Our phylogeny suggests a more complex scenario that includes *S. dunni* from the dry valley of the Mira River in northwestern Ecuador. In any case, the three species are segregated geographically in western Ecuador, with *S. n. leucomelas* occupying the evergreen lowland and forest of northwestern Chocoan Ecuador, *S. bevridgelyi* the semi-deciduous forest in southwestern Ecuador, and *S. dunni* dry montane shrublands. Whether the current low genetic divergence between these three taxa constitutes a scenario of recent or ongoing gene flow between them is worth addressing further using nuclear markers. Strong local selection may have affected traits other than the mitochondrial genes.

Unlike the previous examples, the pattern of cladogenesis recovered in our phylogeny for the species of the *Dipsas peruana* complex (Fig. 3) suggest that a series of allopatric speciation events could be responsible for the current observed pattern of geographic genetic divergence between *D. peruana* and *D. palmeri* + *D. klebbai*. Two geographic barriers (i.e., Napo and Marañón rivers; Fig. 4) are located between the geographic ranges of the aforementioned species, and these features of the Andean geography have previously been recognized as important barriers to gene flow (Hackett 1993, Funk et al. 2007, Lynch Alfaro et al. 2015).

A different scenario of speciation can be interpreted from the current distribution (Fig. 5) of the clade comprised by *Dipsas georgejetti*, *D. oligozonata*, *D. oswaldobaezi*, and *D. williamsi*. All of these species are adapted to dry shrublands, and the distribution of this vegetation type in northern Peru and south-central Ecuador is not continuous. We hypothesize that the discontinuity of dry shrubland west of the Andes in Ecuador and Peru is what explains best the observed pattern of geographic genetic divergence in this group of snakes.

We suspect that there are numerous additional species to be described across all genera of Dipsadini. Our results and the results of other recent researchers such as Sheehy (2012) indicate that additional taxonomic changes are also needed at the species-group and genus level to create a robust, stable taxonomy that agrees with the molecular phylogeny. Other morphological data such as visceral topology (e.g., Wallach 1995) suggest that morphological synapomorphies may exist for these clades, but are complex and difficult to identify accurately. Hence, in order to clarify species richness and higher-level to detailed relationships in Dipsadini, a systematically intensive revision that includes genetic, biogeographic, and morphological data from the greatest number of species representing the known genera is needed.

## Author contributions

Conceived and designed the work: AA JMG DSV OTC. Performed the analyses: AA RAP NP. Gathered morphological data: AA KM GA JCSN TJC RAP DSV DFCH PJV MYM OTC. Contributed reagents/materials/analysis tools: RAP JMG DSV NP GRC PJV TJC DFCH. Wrote the paper: AA DSV KM NP GA JCSN RAP DFCH PJV MYM JMG OTC.

## Supplementary Material

XML Treatment for Sibon bevridgelyi

XML Treatment for Dipsas bobridgelyi

XML Treatment for Dipsas georgejetti

XML Treatment for Dipsas oswaldobaezi

XML Treatment for Dipsas klebbai

XML Treatment for Dipsas palmeri

XML Treatment for Dipsas peruana

XML Treatment for Dipsas latifrontalis

## Appendix 1

**Table:** GenBank accession numbers for loci and terminals of taxa and outgroups sampled in this study. Novel sequence data produced in this study are marked with an asterisk (*).

Species	Voucher	Country	12S	16S	CYTB	ND4	c-mos
*A. iridescens*	MZUTI 4178	Ecuador	-	KT944040	KY610080	-	KT944066
*D. albifrons*	MZUSP 13993	Brazil	JQ598803	JQ598866	JQ598925	-	-
*D. andiana*	MZUTI 3501	Ecuador	-	MH341009*	MH375032*	-	-
*D. andiana*	MZUTI 3505	Ecuador	-	MH341010*	MH374974*	-	-
*D. andiana*	MZUTI 5413	Ecuador	-	MH341011*	MH374978*	-	-
*D. andiana*	QCAZ 10756	Ecuador	-	MH341014*	MH375012*	-	-
*D. andiana*	QCAZ 13538	Ecuador	-	MH341015*	MH375018*	-	-
*D. andiana*	QCAZ 5731	Ecuador	-	MH341012*	MH375005*	-	-
*D. andiana*	QCAZ 8452	Ecuador	-	MH341013*	MH375011*	-	-
*D. articulata*	USNM 348490	Panama	JQ598804	JQ598867	-	-	-
*D. bobridgelyi*	MZUTI 5414	Ecuador	-	MH341016*	MH374984*	-	-
*D. bobridgelyi*	MZUTI 5417	Ecuador	-	MH341017*	MH374985*	-	-
*D. bucephala*	GRCOLLI 25659	Brazil	MH341087*	MH341018*	MH375026*	MH375052*	MH374932*
*D. bucephala*	IBSP72899	Brazil	GQ457789	GQ457730	-	-	GQ457850
*D. catesbyi*	KU 214851	Peru	-	-	EF078537	EF078585	-
*D. catesbyi*	LSUMNS 13989	Brazil	-	KX660267	KX660536	-	-
*D. catesbyi*	MZUSP 14664	Brazil	JQ598805	KX694637	KX694856	-	JQ598977
*D. catesbyi*	QCAZ 13558	Ecuador	MH341088*	MH341019*	MH374975*	MH375042*	MH374933*
*D. elegans*	DHMECN 10311	Ecuador	-	MH341020*	MH374979*	-	-
*D. elegans*	MZUTI 3317	Ecuador	-	MH341021*	MH375033*	-	-
*D. elegans*	MZUTI 3695	Ecuador	-	MH341022*	MH375031*	-	-
*D. elegans*	ZSFQ 10	Ecuador	-	-	MH374994*	-	-
*D. elegans*	ZSFQ 151	Ecuador	-	MH341023*	MH374992*	-	-
*D. ellipsifera*	MZUTI 4931	Ecuador	-	MH341024*	MH375030*	-	MH374934*
*D. ellipsifera*	TH	Ecuador	-	-	MH374966*	-	MH374935*
*D. georgejetti*	MZUA.RE.121	Ecuador	-	MH341025*	MH375024*	-	MH374936*
*D. georgejetti*	MZUA.RE.122	Ecuador	-	MH341026*	MH375025*	-	MH374937*
*D. georgejetti*	QCAZ 10589	Ecuador	-	MH341027*	-	-	-
*D. gracilis*	JMG 070	Ecuador	-	MH341028*	MH374980*	-	MH374938*
*D. gracilis*	MZUTI 1386	Ecuador	-	MH341029*	MH374970*	-	-
*D. gracilis*	MZUTI 3331	Ecuador	-	MH341030*	MH374995*	-	-
*D. gracilis*	MZUTI 3503	Ecuador	-	MH341031*	MH375023*	-	-
*D. gracilis*	QCAZ 10196	Ecuador	-	MH341033*	MH375000*	-	-
*D. gracilis*	QCAZ 11238	Ecuador	-	MH341034*	MH375001*	-	-
*D. gracilis*	QCAZ 12478	Ecuador	-	MH341035*	MH375002*	-	-
*D. gracilis*	QCAZ 15717	Ecuador	-	MH341036*	MH375013*	-	-
*D. gracilis*	QCAZ 5265	Ecuador	-	-	MH374998*	-	-
*D. gracilis*	QCAZ 5886	Ecuador	-	MH341032*	MH374999*	-	-
*D. indica*	-	French Guiana	NN	AF158488	-	-	-
*D. indica ecuadoriensis*	QCAZ 13305	Ecuador	MH341089*	MH341037*	MH375006*	MH375043*	MH374939*
*D. indica ecuadoriensis*	QCAZ 13306	Ecuador	MH341090*	MH341038*	MH375007*	MH375044*	MH374940*
*D. indica ecuadoriensis*	QCAZ 13561	Ecuador	MH341091*	MH341039*	MH375008*	MH375045*	MH374941*
*D. jamespetersi*	AMARU 1123	Ecuador	-	MH341040*	-	-	MH374943*
*D. jamespetersi*	AMARU 383	Ecuador	-	-	-	-	MH374942*
*D. jamespetersi*	CAMPO 488	Ecuador	-	MH341041*	MH375028*	-	MH374944*
*D. jamespetersi*	QCAZ 9190	Ecuador	-	MH341042*	MH375014*	-	-
*D. klebbai*	JMG 050	Ecuador	-	MH341043*	MH375022*	-	MH374945*
*D. klebbai*	MZUTI 5412	Ecuador	-	MH341045*	MH374977*	-	-
*D. klebbai*	MZUTI 63	Ecuador	-	MH341044*	MH374986*	-	-
*D. klebbai*	QCAZ 12717	Ecuador	-	MH341046*	MH375019*	-	-
*D. klebbai*	QCAZ 12799	Ecuador	-	MH341047*	MH374996*	-	-
*D. klebbai*	QCAZ 14280	Ecuador	-	MH341048*	-	-	-
*D. klebbai*	QCAZ 14281	Ecuador	-	MH341049*	-	-	-
*D. mikanii*	MZUSP 14658	Brazil	GQ457832	GQ457771	KX694855	-	GQ457892
*D. neuwiedi*	MCP13291	Brazil	GQ457831	GQ457770	-	-	GQ457891
*D. neuwiedi*	MZUSP 13972	Brazil	JQ598838	JQ598898	-	-	-
*D. oligozonata*	MZUA.RE.081	Ecuador	-	MH341050*	MH375029*	-	-
*D. oreas*	DHMECN 7647	Ecuador	-	MH341051*	MH374971*	-	-
*D. oreas*	DHMECN 7648	Ecuador	-	MH341052*	MH374967*	-	-
*D. oreas*	MZUA.RE.239	Ecuador	-	MH341053*	MH374987*	-	-
*D. oreas*	MZUTI 3351	Ecuador	-	MH341054*	-	MH375038*	-
*D. oreas*	MZUTI 5415	Ecuador	-	MH341055*	-	-	-
*D. oreas*	MZUTI 5418	Ecuador	-	MH341056*	MH374981*	-	-
*D. oreas*	QCAZ 10068	Ecuador	-	MH341057*	MH375015*	-	-
*D. oreas*	QCAZ 11290	Ecuador	-	MH341058*	MH375016*	-	-
*D. oreas*	QCAZ 13875	Ecuador	-	MH341059*	MH375017*	-	-
*D. oswaldobaezi*	QCAZ 10369	Ecuador	-	MH341060*	MH374997*	-	-
*D. palmeri*	JMG 069	Ecuador	-	MH341061*	MH374976*	-	MH374946*
*D. palmeri*	MZUTI 4804	Ecuador	-	MH341062*	MH374982*	-	MH374947*
*D. palmeri*	MZUTI 4975	Ecuador	-	MH341063*	-	-	-
*D. palmeri*	MZUTI 5419	Ecuador	-	MH341064*	MH374988*	-	MH374948*
*D. palmeri*	QCAZ 13304	Ecuador	MH341092*	MH341065*	MH375009*	MH375046*	MH374949*
*D. palmeri*	QCAZ 13307	Ecuador	MH341093*	MH341066*	MH375004*	MH375047*	MH374950*
*D. palmeri*	QCAZ 13562	Ecuador	MH341094*	MH341067*	MH375010*	MH375048*	MH374951*
*D. pavonina*	LSUMNS 14372	Brazil	-	KX660268	KX660537	-	-
*D. pavonina*	MZUTI 4972	Ecuador	-	MH341068*	MH374983*	-	MH374952*
*D. peruana*	LSUMNS 1532	Peru	-	-	KX660538	-	KX660406
*D. pratti*	MHUA 14278	Colombia	-	-	GQ334482	GQ334583	-
*D. temporalis*	QCAZ 5050	Ecuador	-	MH341069*	MH375003*	-	-
*D. turgida*	FML 14969	Argentina	JQ598839	JQ598899	KX660547	-	-
*D. turgida*	LSUMNS 6459	-	-	KX660279	-	KX660659	KX660418
*D. vaga*	KU 219121	Peru	-	KX660252	-	-	KX660393
*D. variegata*	MZUSP 14665	Brazil	-	GQ457731	-	-	GQ457851
*D. variegata*	-	-	AF158406	AF158476	-	-	-
*D. ventrimaculata*	MCP4870	Brazil	JQ598840	JQ598900	-	-	JQ598997
*D. vermiculata*	MZUTI 3663	Ecuador	-	MH341070*	MH374989*	-	-
*D. vermiculata*	QCAZ 13563	Ecuador	MH341095*	MH341071*	MH374972*	MH375049*	MH374953*
*D. vermiculata*	QCAZ 13582	Ecuador	MH341096*	MH341072*	-	MH375040*	MH374954*
*D. vermiculata*	QCAZ 13825	Ecuador	-	MH341073*	MH374973*	MH375050*	MH374955*
*D. vermiculata*	SBI 171139	Peru	Z46459	Z46496	-	-	-
*D. williamsi*	CORBIDI 12695	Peru	-	-	MH374968*	MH375041*	-
*D. williamsi*	CORBIDI 12919	Peru	-	-	MH374969*	MH375039*	-
*G. godmani*	-	-	JQ598814	JQ598877	JQ598932	-	-
*S. annulatus*	ADM 0007	Costa Rica	-	KX660170	KX660444	KX660573	KX660309
*S. annulatus*	ADM 242	Costa Rica	-	KX660169	KX660443	KX660572	KX660308
*S. annulatus*	MVZ 269290	Nicaragua	MH341097*	MH341074*	MH375034*	MH375053*	MH374956*
*S. annulatus*	MZUTI 3034	Ecuador	-	MH341075*	MH375021*	-	-
*S. anthracops*	MVZ 215680	Costa Rica	MH341098*	MH341076*	MH375035*	MH375054*	MH374957*
*S. bevridgelyi*	MZUA.RE.424	Ecuador	-	-	MH374990*	-	-
*S. bevridgelyi*	MZUTI 3269	Ecuador	-	MH341077*	MH374962*	-	-
*S. bevridgelyi*	MZUTI 5416	Ecuador	-	MH341078*	MH374963*	-	-
*S. dimidiatus*	LSUMNS 6689	-	-	KX660278	-	-	KX660417
*S. dunni*	CAMPO 533	Ecuador	-	MH341079*	MH374991*	-	-
*S. longifrenis*	MVZ 215681	Costa Rica	MH341099*	MH341080*	MH375036*	MH375055*	MH374958*
*S. merendonensis*	MVZ 263880	Guatemala	MH341100*	MH341081*	MH375037*	MH375056*	MH374959*
*S. nebulatus hartwegi*	MHUA14511	Colombia	-	-	GQ334556	GQ334662	-
*S. nebulatus leucomelas*	DHMECN 9585	Ecuador	-	MH341082*	-	-	-
*S. nebulatus leucomelas*	MZUTI 3911	Ecuador	-	MH341083*	MH374964*	-	-
*S. nebulatus leucomelas*	MZUTI 4810	Ecuador	-	MH341084*	MH374965*	-	MH374960*
*S. nebulatus nebulatus*	Belize	Belize	AF544777	AF544806	-	-	AF544736
*S. nebulatus nebulatus*	MVZ 233298	Costa Rica	EU728583	EU728583	EU728583	EU728583	-
*T. fasciata*	TJC 666	Mexico	MH341101*	MH341085*	MH375027*	MH375057*	MH374961*
*T. fischeri*	MVZ 143527	Guatemala	MH341102*	MH341086*	MH374993*	MH375051*	-
*T. sartorii*	KU 289806	El Salvador	-	-	EF078540	EF078588	-

## Appendix 2

**Table:** List of PCR and sequencing primers and their respective PCR conditions (denaturation, annealing, extension and number of corresponding cycles) used in this study. All PCR protocols included an initial 3-min step at 94 °C and a final extension of 10 min at 72 °C.

Locus	Primer name	Sequence (5’-3’)	Reference	PCR profile:
16S	16Sar-L	CGCCTGTTTATCAAAAACAT	Palumbi et al. (1991)	30 cycles of 94 °C (45 sec), 53 °C (45 sec), 72 °C (1 min)
	16Sbr-H-R	CCGGTCTGAACTCAGATCACGT		
Cytb	GLUDG-L	TGACTTGAARAACCAYCGTTG	Palumbi et al. (1991)	35–42 cycles of 95°C (30 sec) , 50 or 56 °C (45 sec), 72 °C (45 sec)
	ATRCB3	TGAGAAGTTTTCYGGGTCRTT	Harvey et al. (2000)	
ND4	ND4	CACCTATGACTACCAAAAGCTCATGTAGAAGC	Arévalo et al. (1994)	94 °C (25 sec), 56 or 60 °C (1 min), 72 °C (2 min) [x25–30]
	Leu	CATTACTTTTACTTGGATTTGCACCA		
c-mos	S77	CATGGACTGGGATCAGTTATG	Lawson et al. (2005)	1 cycle of 94 °C (3 min), 56 °C (45 sec), 72 °C (1 min), followed by 34 cycles of 94 °C (45 sec), 56 °C (45 sec), 72 °C (1 min)
	S78	CCTTGGGTGTGATTTTCTCACCT		

## Appendix 3

**Table:** Morphological data and sex for specimens of Dipsadini species examined. Codes: V = ventrals; SC = subcaudals; D1–3 = dorsal scale rows at neck, midbody, and vent; PO = postoculars; SL = supralabials; IL = infralabials; SVL = snout-vent length (mm); TL = tail length (mm); M = Male, F = Female.

Species	Voucher	V	SC	D1	D2	D3	PO	SL	IL	SVL	TL	Sex
*D. andiana*	MZUA.RE.0230	187	96	15	15	15	3	9	11	744	196	M
*D. andiana*	MHNG 2250.053	194	85	15	15	15	2	9	12	292	71	F
*D. andiana*	MZUTI 5413	190	101	14	15	15	2	10	11	471	165	M
*D. andiana*	MZUTI 3501	187	98	15	15	15	2	9	12	398	137	M
*D. andiana*	MZUTI 3505	192	–	15	15	15	2	8	10	674	167	F
*D. andiana*	ZSFQ D115	189	84	15	15	15	2	10	10	680	150	F
*D. andiana*	ZSFQ D116	186	90	15	15	15	2	10	10	453	149	M
*D. andiana*	ZSFQ D117	189	101	15	15	15	2	9	9	405	139	M
*D. bobridgelyi*	QCAZ 1706	201	117	15	15	15	2	9	12	445	212	M
*D. bobridgelyi*	DHMECN 11527	178	98	15	15	15	2	9	12	404	158	F
*D. bobridgelyi*	MZUTI 3266	184	96	15	15	15	2	9	11	286	117	F
*D. bobridgelyi*	MZUTI 5414	180	95	15	15	15	2	9	13	478	195	M
*D. bobridgelyi*	MZUTI 5417	182	101	15	15	15	2	9	13	372	158	M
*D. catesbyi*	MHNG 2220.054	180	98	13	13	13	1	8	9	366	147	F
*D. catesbyi*	MHNG 2238.005	176	94	13	13	13	2	9	10	420	155	F
*D. catesbyi*	USNM 283949	168	81	13	13	13	1	7	8	276	98	F
*D. catesbyi*	DHMECN 11555	164	97	–	–	–	2	7	–	222	80	–
*D. catesbyi*	QCAZ 181	172	93	13	13	13	2	9	10	470	169	F
*D. catesbyi*	MHNG 2220.052	175	83	13	13	13	1	8	10	505	180	F
*D. catesbyi*	QCAZ 210	199	97	13	13	13	2	8	9	441	165	F
*D. catesbyi*	MHNG 2206.086	183	108	13	13	13	1	8	9	480	202	M
*D. catesbyi*	MHNG 2435.097	184	98	13	13	13	1	8	10	308	117	F
*D. catesbyi*	QCAZ 5108	197	105	13	13	13	2	7	8	583	223	M
*D. catesbyi*	MHNG 2249.001	178	93	13	13	13	2	8	9	311	112	F
*D. catesbyi*	QCAZ 28	181	101	13	14	13	2	8	10	591	232	F
*D. catesbyi*	MHNG 2238.014	175	93	13	13	13	2	8	9	429	158	F
*D. catesbyi*	MHNG 2307.091	181	92	13	13	13	2	7	9	431	155	F
*D. catesbyi*	MZUTI 4736	187	103	13	13	13	2	8	10	454	186	M
*D. catesbyi*	MZUTI 4999	177	93	13	13	13	2	9	9	397	160	M
*D. elegans*	MHNG 2435.084	178	102	15	15	15	2	6	10	305	111	M
*D. elegans*	MHNG 2440.098	180	98	15	15	15	2	7	11	178	65	F
*D. elegans*	DHMECN 1693	181	97	15	15	15	2	7	9	119	71	M
*D. elegans*	MHNG 2457.078	182	94	15	15	15	1	7	10	183	65	F
*D. elegans*	MHNG 2249.019	179	95	15	15	15	2	6	11	505	195	M
*D. elegans*	MHNG 2413.074	178	93	15	15	15	1	7	11	607	211	F
*D. elegans*	USNM 285957	183	100	15	15	13	2	7	11	152	60	–
*D. elegans*	MHNG 2399.072	180	82	15	15	15	1	7	9	555	161	F
*D. elegans*	MZUTI 3695	182	102	15	15	15	2	7	11	296	102	M
*D. elegans*	MZUTI 3317	186	108	15	15	15	2	7	11	409	175	M
*D. elegans*	MHNG 2457.079	177	90	15	15	15	2	7	9	687	246	F
*D. elegans*	MHNG 2308.002	180	90	15	15	13	2	7	10	605	212	F
*D. elegans*	MHNG 2220.093	181	109	15	15	15	1	8	10	591	251	M
*D. elegans*	MZUTI 3316	182	86	15	15	15	1	8	11	657	220	F
*D. ellipsifera*	MZUTI 4931	164	86	15	15	15	2	7	10	229	79	M
*D. ellipsifera*	QCAZ 14855	175	93	15	15	15	2	8	7	580	230	M
*D. ellipsifera*	QCAZ 15225	183	101	15	15	15	2	6	8	488	234	M
*D. ellipsifera*	MHNG 2220.048	163	62	15	15	15	2	7	8	406	114	F
*D. gracilis*	QCAZ 4137	185	94	15	15	15	2	8	9	289	128	M
*D. gracilis*	QCAZ 14495	179	99	15	15	15	2	10	11	530	235	F
*D. gracilis*	QCAZ 7321	189	98	15	15	15	2	11	10	361	150	M
*D. gracilis*	MZUA.RE.0280	189	101	13	13	13	3	10	12	590	172	F
*D. gracilis*	MZUA.RE.0281	184	110	15	15	15	2	10	12	343	100	–
*D. gracilis*	QCAZ 12478	193	102	15	15	15	2	11	10	425	161	M
*D. gracilis*	MHNG 2309.038	190	–	15	15	15	2	8	11	291	92	M
*D. gracilis*	QCAZ 10196	180	89	15	15	15	2	10	12	283	110	M
*D. gracilis*	USNM 285477	203	118	15	15	15	3	9	13	356	166	M
*D. gracilis*	USNM 285478	197	118	15	15	15	3	9	12	416	189	–
*D. gracilis*	USNM 285479	203	131	15	15	15	3	9	12	418	199	M
*D. gracilis*	USNM 285480	193	107	15	15	15	2	10	11	181	74	–
*D. gracilis*	DHMECN 2902	205	121	15	15	15	2	7	11	554	265	M
*D. gracilis*	QCAZ 11427	196	–	15	15	15	2	9	11	365	150	M
*D. gracilis*	MHNG 1363.023	206	120	15	15	15	3	11	11	395	187	M
*D. gracilis*	MHNG 1363.024	210	122	15	15	14	3	10	12	458	210	M
*D. gracilis*	MHNG 1363.026	201	113	15	15	15	2	9	12	464	203	F
*D. gracilis*	MHNG 1363.027	209	118	15	15	15	2	10	11	426	193	M
*D. gracilis*	MHNG 2453.019	203	113	15	15	15	2	9	11	332	142	F
*D. gracilis*	QCAZ 14494	203	109	15	15	15	2	7	8	473	220	M
*D. gracilis*	MZUTI 1386	194	110	15	15	15	2	9	12	451	187	F
*D. gracilis*	MZUTI 3503	197	113	15	15	15	2	9	10	468	199	F
*D. gracilis*	DHMECN 129	192	101	15	15	15	2	9	11	425	191	M
*D. gracilis*	MZUTI 4199	205	109	15	15	15	2	10	11	402	166	F
*D. indica*	MZUA.RE.0059	180	97	13	13	13	2	9	15	1153	293	F
*D. indica*	MHNG 2435.093	196	95	13	13	11	2	9	14	537	172	F
*D. indica*	MHNG 2413.076	197	109	13	13	11	2	9	14	327	121	M
*D. indica*	MZUTI 4735	199	112	13	13	13	2	9	14	672	230	M
*D. jamespetersi*	MZUA.RE.0147	178	71	15	15	15	2	8	13	663	156	M
*D. jamespetersi*	MZUTI 5307	178	75	15	15	15	2	7	11	560	156	H
*D. jamespetersi*	USNM 237040	183	84	15	15	15	2	8	11	150	61	–
*D. jamespetersi*	MHNG 2512.047	171	81	15	15	15	2	8	9	424	152	M
*D. jamespetersi*	MHNG 2512.048	186	76	15	15	15	2	8	12	511	157	F
*D. jamespetersi*	MHNG 2399.071	178	73	15	15	15	2	8	10	469	136	F
*D. jamespetersi*	MHNG 2457.09	179	69	15	15	15	2	8	11	–	–	F
*D. jamespetersi*	MHNG 2512.049	169	83	15	15	15	3	8	10	421	154	M
*D. jamespetersi*	MHNG 2512.05	190	–	15	15	15	2	8	9	466	125	F
*D. jamespetersi*	MHNG 2521.087	178	81	15	15	15	3	8	9	378	133	M
*D. jamespetersi*	QCAZ 15100	185	98	15	15	15	2	8	7	412	133	M
*D. jamespetersi*	MHNG 2413.082	185	73	15	15	15	2	8	11	505	143	F
*D. klebbai*	QCAZ 1605	181	97	15	15	15	2	9	10	569	251	M
*D. klebbai*	DHMECN 568	–	104	15	15	15	2	10	13	630	240	F
*D. klebbai*	MHNG 2220.035	194	115	15	15	15	2	11	14	286	118	F
*D. klebbai*	MHNG 2220.056	185	106	13	13	13	2	8	8	505	209	M
*D. klebbai*	MHNG 2250.063	197	104	15	15	15	2	9	12	489	199	M
*D. klebbai*	MHNG 2250.064	196	109	15	15	15	2	10	12	401	169	M
*D. klebbai*	MZUTI 5412	188	116	15	15	15	2	10	14	608	262	M
*D. klebbai*	USNM 286323	198	117	15	15	15	2	8	11	263	105	F
*D. klebbai*	MHNG 2220.038	193	106	15	15	15	2	9	11	570	219	F
*D. klebbai*	MHNG 2220.039	190	109	15	15	15	2	11	12	500	204	M
*D. klebbai*	MZUTI 63	199	112	15	15	15	2	10	12	701	297	M
*D. klebbai*	MHNG 2220.04	191	102	15	15	15	2	9	13	525	210	F
*D. klebbai*	MHNG 2220.041	187	101	15	15	15	2	9	13	604	229	F
*D. klebbai*	QCAZ 250	184	99	15	15	15	2	9	12	495	205	M
*D. klebbai*	QCAZ 14281	201	123	15	15	15	2	9	11	749	330	M
*D. klebbai*	MHNG 2529.029	188	98	15	15	15	2	9	12	534	209	F
*D. klebbai*	ZSFQ D304	189	101	15	15	15	2	9	11	303	121	F
*D. georgejetti*	USNM 142595	182	59	15	15	15	2	7	11	131	30	M
*D. georgejetti*	MZUTI 5411	178	69	15	16	15	2	7	9	315	87	M
*D. georgejetti*	DHMECN 11639	172	86	15	15	15	2	7	10	270	90	M
*D. georgejetti*	MZUA.RE.0121	177	58	15	15	15	2	7	10	856	150	F
*D. georgejetti*	MZUA.RE.0122	175	79	15	15	15	2	7	9	711	170	M
*D. georgejetti*	DHMECN 11646	180	72	15	15	15	2	7	9	382	140	M
*D. georgejetti*	ZSFQ D606	182	63	15	15	15	2	7	10	163	51	M
*D. oligozonata*	MZUA.RE.0081	144	62	15	15	15	2	8	10	446	127	M
*D. oligozonata*	MZUA.RE.0240	150	53	15	15	15	2	7	12	772	154	F
*D. oligozonata*	MZUA.RE.0020	149	60	15	15	15	2	8	10	632	134	M
*D. oligozonata*	MZUA.RE.0357	138	63	15	15	15	2	8	11	538	139	M
*D. oreas*	DHMECN 7647	168	77	15	15	15	2	7	11	270	106	M
*D. oreas*	DHMECN 7666	170	79	15	15	15	2	8	12	532	107	M
*D. oreas*	MZUA.RE.0239	171	84	15	15	15	2	7	11	785	214	M
*D. oreas*	MZUA.RE.0290	182	95	15	15	15	2	7	13	561	138	–
*D. oreas*	QCAZ 9190	181	93	15	15	15	2	7	10	495	191	F
*D. oreas*	USNM 62797	180	77	15	15	15	2	8	11	377	124	F
*D. oreas*	USNM 62798	178	75	–	15	15	–	–	11	367	120	M
*D. oreas*	USNM 62800	180	84	15	15	15	2	8	12	144	46	M
*D. oreas*	DHMECN 10785	198	97	15	15	15	2	7	11	222	76	M
*D. oreas*	DHMECN 2572	178	89	15	15	15	2	7	11	417	141	M
*D. oreas*	MZUTI 3351	177	87	14	15	15	2	7	10	473	181	M
*D. oreas*	MZUTI 5415	178	75	15	15	15	2	7	11	487	156	M
*D. oreas*	MZUTI 5418	183	86	15	15	15	2	7	10	252	81	M
*D. oreas*	MHNG 2514.028	176	–	15	15	15	2	7	11	384	128	M
*D. oreas*	MHNG 2521.084	171	79	15	15	15	2	9	12	571	184	F
*D. oreas*	QCAZ 10068	157	84	15	15	15	2	8	11	181	60	M
*D. oreas*	QCAZ 13875	175	63	15	15	15	2	7	10	499	151	M
*D. oreas*	QCAZ 11290	176	102	14	14	14	2	9	12	334	131	M
*D. oreas*	QCAZ 6020	167	59	15	15	15	2	7	–	586	149	M
*D. palmeri*	QCAZ 11411	192	107	15	15	15	2	9	12	470	193	M
*D. palmeri*	QCAZ 5609	193	–	15	15	15	2	9	13	753	271	M
*D. palmeri*	QCAZ 13307	186	118	15	15	15	2	8	11	660	278	M
*D. palmeri*	QCAZ 13562	189	91	15	15	15	2	9	11	215	78	M
*D. palmeri*	QCAZ 4710	182	97	15	15	15	2	9	12	472	200	M
*D. palmeri*	AMNH 24126	196	116	15	15	15	2	9	13	234	91	M
*D. palmeri*	MZUTI 4804	190	101	15	15	15	3	8	10	602	269	M
*D. palmeri*	MZUA.RE.0044	181	86	15	15	15	2	9	10	893	225	F
*D. palmeri*	QCAZ 14071	187	101	15	15	15	2	9	11	642	246	F
*D. palmeri*	QCAZ 3288	185	103	15	15	15	2	9	11	615	274	M
*D. palmeri*	MZUTI 3956	202	116	15	15	15	2	10	12	656	239	M
*D. palmeri*	AMNH 37939	190	–	15	15	15	2	9	13	694	188	M
*D. palmeri*	QCAZ 13992	192	97	15	15	15	2	9	11	–	–	M
*D. palmeri*	QCAZ 4564	185	109	15	15	15	2	9	11	248	93	M
*D. palmeri*	QCAZ 6021	196	103	15	15	15	2	9	12	791	290	M
*D. palmeri*	QCAZ 14338	172	110	15	15	15	2	9	12	907	390	M
*D. palmeri*	QCAZ 12771	197	104	15	15	15	2	9	12	273	98	M
*D. palmeri*	MZUTI 4971	196	106	15	15	15	2	8	12	332	121	M
*D. palmeri*	MZUTI 4975	191	114	15	15	15	2	8	11	631	299	M
*D. palmeri*	QCAZ 12772	182	101	15	15	15	2	9	12	403	146	M
*D. palmeri*	MZUTI 5419	189	117	15	15	15	2	9	13	658	286	M
*D. palmeri*	QCAZ 12510	182	93	15	15	15	2	9	12	740	252	F
*D. palmeri*	MZUA.RE.0119	200	102	15	15	15	2	9	12	1187	298	F
*D. pavonina*	MZUA.RE.0198	211	119	13	13	13	3	11	13	745	241	M
*D. pavonina*	QCAZ 5554	187	95	13	13	13	2	10	12	493	256	M
*D. pavonina*	MHNG 2309.039	204	–	13	13	13	2	9	11	341	90	M
*D. pavonina*	MHNG 2521.088	198	117	13	13	13	2	10	12	413	196	M
*D. pavonina*	MZUTI 4972	196	115	13	13	13	2	9	10	441	207	M
*D. peruana*	AMNH 147037	187	111	15	15	15	2	9	13	199	85	M
*D. peruana*	USNM 60718	177	75	15	15	15	3	9	12	258	79	M
*D. peruana*	USNM 299232	188	92	15	15	15	2	9	13	467	198	M
*D. peruana*	USNM 299233	200	127	15	15	15	2	9	12	235	99	M
*D. oswaldobaezi*	MZUA.RE.0286	185	61	15	15	15	2	6	9	395	70	–
*D. oswaldobaezi*	QCAZ 10369	179	70	15	15	15	2	6	9	277	85	M
*D. oswaldobaezi*	QCAZ 14051	175	66	15	15	15	2	6	7	287	86	–
*D. oswaldobaezi*	QCAZ 14060	180	64	15	15	15	2	6	8	500	132	–
*D. oswaldobaezi*	QCAZ 15108	179	65	15	15	15	2	6	9	407	110	F
*D. temporalis*	MZUTI 3331	202	113	15	15	15	2	9	11	226	92	F
*D. temporalis*	MHNG 2521.083	175	116	15	15	15	1	7	9	234	111	F
*D. vagrans*	AMNH 63373	153	82	15	15	15	2	9	11	302	129	M
*D. vermiculata*	MHNG 2521.085	181	93	15	15	15	–	–	–	215	81	F
*D. vermiculata*	DHMECN 11197	181	116	13	13	13	2	8	9	109	84	M
*D. vermiculata*	MHNG 2436.014	182	103	13	13	13	2	9	8	270	104	M
*D. vermiculata*	MZUTI 5080	183	113	13	13	13	2	7	9	515	220	M
*D. vermiculata*	QCAZ 13825	191	116	15	15	15	2	8	9	588	279	M
*D. vermiculata*	MZUTI 4738	183	105	13	13	13	2	7	9	234	90	M
*D. vermiculata*	MZUTI 3663	181	98	13	13	13	2	8	9	542	195	M
*D. vermiculata*	MZUA.RE.0261	183	107	13	13	13	1	8	9	689	190	M
*S. annulatus*	MZUTI 3034	197	–	15	15	14	2	7	8	464	70	M
*S. bevridgelyi*	AMNH 22092	185	88	15	15	15	2	7	10	572	268	M
*S. bevridgelyi*	MZUA.RE.0424	181	82	15	15	15	2	7	8	515	126	M
*S. bevridgelyi*	QCAZ 14446	181	94	15	15	15	2	8	7	545	220	M
*S. bevridgelyi*	QCAZ 14444	179	80	15	15	15	2	7	8	487	156	M
*S. bevridgelyi*	MZUA.RE.0142	193	98	15	15	15	2	7	7	786	204	F
*S. bevridgelyi*	DHMECN 9483	182	91	15	15	15	2	7	11	436	158	M
*S. bevridgelyi*	MZUTI 3269	175	88	15	15	15	2	7	9	349	124	M
*S. bevridgelyi*	MZUTI 5416	184	80	15	16	15	2	7	9	602	186	M
*S. bevridgelyi*	ZSFQ D503	182	87	15	15	15	2	7	9	405	122	M
*S. nebulatus*	MZUTI 4810	187	–	15	15	15	2	7	7	480	110	F
*S. nebulatus*	MZUTI 3911	186	67	15	15	15	2	7	9	280	90	M
*S. nebulatus*	USNM 285501	184	95	15	15	15	2	7	8	363	127	M
*S. nebulatus*	MZUA.RE.0328	183	95	15	15	15	2	8	10	732	190	M
*S. nebulatus*	MZUA.RE.0174	178	78	15	15	15	2	7	9	714	170	F
*S. nebulatus*	USNM 285498	184	80	15	15	15	2	8	10	235	79	–
*S. nebulatus*	USNM 285499	189	90	15	15	15	2	7	9	267	101	M
*S. nebulatus*	USNM 285500	183	95	15	15	15	2	7	9	316	124	–
*S. nebulatus*	MZUTI 5342	185	103	15	15	15	2	7	7	324	104	M
*S. nebulatus*	DHMECN 10061	198	89	15	15	15	2	7	11	447	143	M
*S. nebulatus*	USNM 283534	183	94	15	15	15	2	7	9	501	185	–
